# Comparison of the Genetic Structure of Invasive Bigheaded Carp (*Hypophthalmichthys* spp.) Populations in Central-European Lacustrine and Riverine Habitats

**DOI:** 10.3390/ani11072018

**Published:** 2021-07-06

**Authors:** Tamás Molnár, István Lehoczky, Erika Edviné Meleg, Gergely Boros, András Specziár, Attila Mozsár, Zoltán Vitál, Vilmos Józsa, Wahiba Allele, Béla Urbányi, Fatema Ali Al Fatle, Balázs Kovács

**Affiliations:** 1 National Centre for Biodiversity and Gene Conservation, Institute for Farm Animal Gene Conservation, H2100 Gödöllő, Hungary; meleg.erika@nbgk.hu; 2 Institute of Aquaculture and Environmental Safety, Hungarian University of Agriculture and Life Sciences, H2100 Gödöllő, Hungary; Mozsar.Attila@uni-mate.hu (A.M.); Vital.Zoltan@uni-mate.hu (Z.V.); Jozsa.Jozsef.Vilmos@uni-mate.hu (V.J.); wahibaspa@gmail.com (W.A.); Urbanyi.Bela@uni-mate.hu (B.U.); Kovacs.Balazs@uni-mate.hu (B.K.); 3 Centre for Ecological Research, Balaton Limnological Institute, H-8237 Tihany, Hungary; boros.gergely@ecolres.hu (G.B.); specziar.andras@ecolres.hu (A.S.); 4 Doctoral School of Biological Sciences, Hungarian University of Agriculture and Life Sciences, H-2100 Gödöllő, Hungary; fatomtejasmines@yahoo.com

**Keywords:** bigheaded carps, hybridization, genetic variability, invasiveness

## Abstract

**Simple Summary:**

Bigheaded carps (bighead carp and silver carp) originated in Southeast and East Asia, and their hybrids were stocked for economic reasons to Hungarian natural waters such as Lake Balaton for decades, while the Tisza River was populated by escaped individuals with farm origins. The presence of these alien species and their hybrids in Hungarian natural water bodies may pose significant ecological risks (connected with their phytoplankton and zooplankton consumption). To be able to deal with the ecological risks and to understand the potential of invasiveness of these species, one must have information on the population-level genetic structures of these alien fish stocks. Ten microsatellite DNA markers and one mitochondrial marker were used to address these questions. The results showed that the two stocks are genetically different; the lake population was genetically more diverse and consisted of hybrid and silver carp individuals, while the river population contained only silver carps. The mitochondrial sequences found in the two populations originated from the Yangtze River. Based on the different genetic structures of the stocks, one can assume that bigheaded carps do not reproduce in Lake Balaton, while the Tisza River stock represents significant reproductive potential and may become invasive in this river.

**Abstract:**

Bigheaded carps (bighead carp, *Hypophthalmichthys molitrix*, and silver carp, *Hypophthalmichthys nobilis*) and their hybrids play an important ecological and economic role in their original habitat, while their introduced stocks may pose serious ecological risks. To address questions about the persistence and invasiveness of these fish, we need to better understand their population structures. The genetic structures of bigheaded carp populations inhabiting Lake Balaton and the Tisza River were examined with ten microsatellite markers and a mitochondrial DNA marker (COI). The Lake Balaton stock showed higher genetic diversity compared with the Tisza River stock. Based on hierarchical clustering, the Tisza population was characterized only by only silver carps, while the Balaton stock included hybrid and silver carp individuals. All COI haplotypes originated from the Yangtze River. Based on the high genomic and mitochondrial diversity, along with the significant deviation from H–W equilibrium and the lack of evidence of bottleneck effect, it can be assumed that bigheaded carps do not reproduce in Lake Balaton. The present stock in Balaton may have originated from repeated introductions and escapes from the surrounding fishponds. The Tisza stock consists solely of silver carp individuals. This stock appears to have significant reproductive potential and may become invasive if environmental factors change due to climate change.

## 1. Introduction

The silver carp (*Hypophthalmichthys molitrix*) and the bighead carp (*Hypophthalmichthys nobilis*), collectively known as bigheaded carps or filter-feeder Asian carps, have a significant economic role in freshwater fish production. In order to understand the importance of these species, it is sufficient to look at fish harvest reports. The global production of silver carp, for example, was 4,822,794 tons in 2018, putting this species in second place in the world’s aquaculture. The leading producer countries are China, India, Bangladesh, and Iran. Bighead carp ranks fifth among all cultured freshwater fish globally, with a harvested mass of 3,146,466 tons in 2019, accounting for 7.5% of global freshwater aquaculture production [1]. Bigheaded carps are produced in Hungary as part of the so-called “carp polyculture system” and represented about 7–10% of the total fish production of the country in recent years, with a total harvested mass of 1.369 tons in 2019 [2]. The total European production was 39,158 tons in the same year [1]. In addition, these planktivorous Cyprinids play an important ecological role in their original habitat (Eastern Asia) and in the approximately 80 countries they have been introduced to. Because of their high invasive potential, the presence of bigheaded carps is considered an ecological threat in many areas outside of their native range. Bighead carp have already been introduced into 74 countries and are reproducing in 19, while silver carp have been introduced into 88 countries and are successfully reproducing in 23 [3]. Their presence, especially in the United States, has led to serious ecological problems. Significant dominance has been achieved in the Mississippi River’s watershed, which means a biomass proportion in the total fish stock of up to 60% in some locations [4,5].

In the European Union, bigheaded carps have not yet been included in the list of invasive species [6], as their introduction into Western Europe was small-scale, and little attention has been paid so far to the potential reproductive capacity of the stocks [7]. According to the regulation issued by the EU [8], a property of invasive alien species is that their stocks “threaten or adversely impact upon biodiversity and related ecosystem services”. In the case of bigheaded carps, the establishment, distribution, and ecological effects of individual populations are highly dependent on local habitat conditions. The ecological impact of bigheaded carps in North America is already well known (reviewed by [9]), but only a few studies are available from European natural waters. However, the environmental disaster caused by cyanide pollution in the Tisza River in 2000 pointed out the potential of the species to cause/pose an ecological danger. Bigheaded carps have never been stocked into this river (or at least there were no documented stockings), but seven years after the pollution event, 60% of the biomass of the fish stock in Tisza-tó (also called Kisköre reservoir, a 127 km^2^ artificial waterbody constructed as part of the Tisza River flood control project in 1973) was made up of invasive species, and the biomass of Bigheaded carps was 10.5% of the total biomass [10]. These fish originated from individuals that escaped from aquaculture operations located in the catchment area of the river, which subsequently reproduced in the reservoir. In a different study, the feeding habits of hybrid bigheaded carps were examined in Lake Balaton [11]. This is a large shallow lake in Central Europe inhabited by a considerable bigheaded carp stock. Between 1972 and 1983, about 1.5 million bigheaded carp individuals (most likely hybrids) were introduced to the lake [12]. No further introduction has happened since 1983, but there is still a large number of mature individuals living in the lake. The primary food for this fish, which accounted for approximately 20–30% of the total fish stock during the late 1990s and the beginning of the 2000s, was zooplankton in the oligo-mesotrophic lake, which may have a substantial impact on the entire ecosystem of Lake Balaton.

The two main factors responsible for the successful reproduction of bighead carp and silver carp are optimal temperature and water flow. Regarding temperature, the two species require a minimum temperature of 18 °C to reproduce, which is not a limiting factor in most of Europe. Bigheaded carps require intense water currents in the spawning grounds for egg maturation to occur, while silver carp females are less demanding in this regard [13]. In Europe, the introduction of these species began in the 1960s; however, in Western Europe, potential reproduction was not taken into account [14]. The first proven reproduction for bighead carp was reported in the Po River and its associated canal system [7].

However, the situation in Europe is further complicated by the use of the two species (and their hybrids) in aquaculture production as an integral part of the so-called “carp polyculture system” under pond farming conditions, which is a common practice in Central and Eastern Europe [15]. In this region, the environmental conditions required for spawning are given in the Danube and Tisza Rivers basins. There are reports on spawning observations of the two species in the Romanian and Serbian sections of the Danube and the Tisza Rivers [16,17,18]. In Lake Balaton, the spawning could occur occasionally, but the hatching and survival of the fry are not confirmed [19].

Examination of genetic diversity can provide information on the adaptability, persistence, and invasiveness of a species under the given conditions. High genetic diversity often implies better adaptability, and multiple introductions from different sources also influence the control of the invasive species. The difference in the genetic structure of the native and introduced populations (reviewed by [20]) suggests that the interaction of the genetic background and the ecological environment has a significant role in the adaptation of the species and influence the choice of the proper management techniques on the individual habitats. In North America, a study on the genetic structure of bigheaded carps [21] showed that they could expand rapidly, even with lower diversity, compared with Asian stocks, as indicated by the lack of structure. The available information is limited on the genetic structure of the European silver carp and bighead carp stocks. Examination of the mitochondrial genome of the silver carp showed lower nucleotide and haplotype diversity in the Danube stocks compared with the original Asian stocks and higher diversity compared with the Mississippi stocks. However, unlike the Mississippi population, the Danube stock did not show a marked genetic difference from the native populations (Yangtze and Amur Rivers) [22]. In contrast to the American and Asian stocks, microsatellite data on the European populations are missing. Only basic genetic information on the Balaton stock (observed and expected heterozygosity from 10 loci) has been published [19].

This study aimed to perform a genetic comparison between two Central European stocks of the bigheaded carps in order to explore the genetic structure between and within lacustrine and riverine habitats.

## 2. Materials and Methods

### 2.1. Sample Collection, DNA Extraction, Microsatellite Analysis

**Figure 1:**
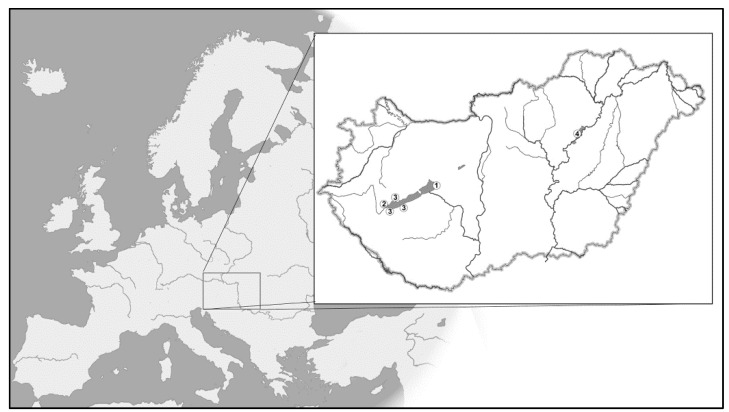
Map of sample locations. Locations 1 and 2, Balaton stock; location 3, inflow stock; location 4, Tisza River stock.

The sampling locations are shown in Figure 1. In the case of Lake Balaton, we used 108 fin samples collected in the study [19] between 2011 and 2013 describing the species hybrid’s reproductive status for further analysis, supplemented with an additional eight samples collected from the inflow areas of the lake. A further 31 samples were collected from the Tisza River (at the Tisza-tó section) in 2014. Since we assumed that the stocks in these habitats comprised of hybrids of the two species, pure silver carp (*n* = 20) and bighead carp (*n* = 21) samples from a gene bank (reference populations at the Northwest Fisheries Resource Development and Management Project (NFRDMP), Parbatipur, Bangladesh) were also used as controls in the study. DNA was extracted using the Qiagen DNeasy^®^ Blood and Tissue Kit (Qiagen GmbH, Hilden, Germany) following the extraction protocol outlined by the manufacturer.

A total of ten cross-species autosomal microsatellite markers [23] were used to genotype all 180 Asian carp samples. Amplification was carried out in a 15 μL reaction volume, and the polymerase chain reaction was conducted with Dream Taq (Thermo Fisher Scientific, Waltham, MA, USA), which has a 1× buffer containing 10 mM Tris^®^ HCl (pH 9.0) and 50 mM potassium chloride (KCl). The final reaction conditions were as follows: 1× PCR buffer, 1.5–2.5 mM MgCl_2_, 200 µM of each 2′ deoxynucleotide triphosphate (dNTP), 10 pmol of each of the forward and reverse primer, 1 unit (U) Taq DNA polymerase, and 10–20 ng genomic DNA template. PCR conditions included a denaturation step of 3 min at 94 °C; 5 cycles of 30 s at 94 °C, 30 s at 55 °C, 30 s at 72 °C; 5 cycles of 30 s at 94 °C, 30 s at 52 °C, 30 s at 72 °C; 30 cycles of 30 s at 94 °C, 30 s at 46 °C, 30 s at 72 °C; and a final extension of 30 min at 72 °C. Amplified fragments were analyzed on an ABI3130 Automatic Fragment Analyzer (Applied Biosystems, Foster City, CA, USA) using POP7 polymer, a 50 cm capillary array, and GS500-LIZ as an internal standard. Fragment length data were analyzed by Genescan and Genotyper 4.0 softwares. (Applied Biosystems, Foster City, CA, USA).

### 2.2. Sequencing of the Mitochondrial COI Region

For the sequence analyses, the cytochrome oxidase C subunit I gene (COI) was amplified with the universal primers for fish barcoding CO1_FF2d_F (TTCTCCACCAACCACAARGAYATYGG) and CO1_FR1d_R (CACCTCAGGGTGTCCGAARAAYCARAA). The PCR master mix contained 1× PCR buffer with (NH_4_)_2_SO_4_ (Fermentas; Thermo Fisher Scientific, Waltham, MA, USA), 0.8 mM dNTP mix, 250 nM for each primer, 2 mM MgCl_2_, 100 ng template DNA, and 1 U Taq polymerase (Fermentas) in a 25 µL final volume. The PCR reactions were performed in an ABI 2720 Thermal Cycler (Applied Biosystems, Foster City, CA, USA) with the following cycling conditions: preliminary denaturation at 95 °C for 2 min, then 30 s at 94 °C, 20 s at 52 °C, and 1 min at 72 °C for 35 cycles. Final elongation was 10 min at 72 °C. PCR products were purified by NucleoSpin Gel and PCR Clean-up Kit (Macherey-Nagel, Düren, Germany). The quality of the purified products was assessed on 1.5% agarose gel run in TBE buffer, then sequenced using the Big Dye Terminator v. 3.1 Cycle Sequencing Kit (Applied Biosystem). The sequences were detected on the ABI 3130 sequencer (Applied Biosystems) with a POP7 polymer and a 50 cm long capillary array.

### 2.3. Statistical Analysis

The presence of null alleles and correction was evaluated using MICRO-CHECKER version 2.2.3 (The University of Hull, Hull, UK) (number of randomizations: 1000, 95% CI) [24]. The allele numbers, unbiased expected and observed heterozygosity, and F were calculated by GenAlEx 6.5 software (The Australian National University, Canberra, Australia) [25]. We estimated the allelic richness and private allelic richness using the rarefaction procedure with the software HP-RARE 1.0 (Montana State University, Bozeman, MT, USA) [26]. The values of Ho and Fis were standardized for the population sizes using weighted means in comparisons between the populations. The comparison of the indices of genetic variability was performed by a Mann–Whitney U-test using Bonferroni correction (with a significance level of 0.01) (SPSS for Windows 11.5 (SPSS Inc.: Chicago, IL, USA) [27]. Analysis of deviations from the Hardy–Weinberg equilibrium (HWE) applying a Markov chain exact test (dememorization number: 5000, number of batches: 500, number of iterations per batch: 5000) [28] for each locus in each population was performed using GENEPOP on the internet [29,30].

The pairwise F_st_ of Weir and Cavalli-Sforza and Edwards genetic distance was calculated using FreeNA software (INRA, Montpellier, France) [31], both with and without ENA correction for the F_st_ and the INA correction for genetic distance. The ENA method corrects for the positive bias induced by the presence of null alleles on Weir’s F_st_ estimation, and INA gives biased estimates of the Cavalli-Sforza and Edwards (1967) genetic distance. The number of replicates was 10,000 for the computation of the bootstrap 95% confidence intervals.

The effective population size was calculated in NeEstimator 2.1 (Molecular Fisheries Laboratory, University of Queensland, Brisbane, Australia) [32] using the linkage disequilibrium method for all natural populations. We also tested for recent and major reductions in population size using BOTTLENECK 1.2.02 (INRA, Montpellier, France) [33]. Significance was tested with the Wilcoxon signed-rank test under a two-phase mutation model (TPM).

The Bayesian algorithm implemented in the software STRUCTURE (University of Chicago, Chicago, IL, USA) [34,35] was used to infer population structure. For estimation, the most probable cluster number K, posterior probabilities (highest lnP(D)), and the ΔK method of [36] were calculated using STRUCTURE HARVESTER software (University of California, Santa Cruz, CA, USA) [37]. For assessing the number of population clusters, an admixture scenario with allele frequencies correlated was chosen; the burn-in was set to 10^4^ and the number of further MCMC runs was set to 10^5^. Calculations were repeated 10 times for each K.

DNA sequences were edited and aligned using Mega-X 10.1 software (The Pennsylvania State University, University Park, TX, USA). The revised alignment was 552 bp for the COI gene. The number of polymorphic (segregating) sites, the total number of mutations, haplotype diversity, and nucleotide diversity were estimated by DnaSP 5.10. software (University of Barcelona, Barcelona, Spain) [38]. A haplotype network for the two populations (Balaton and Tisza) was generated using NETWORK 10.0 software (Fluxus Technology Ltd., Colchester, England) [39]. All haplotypes were blasted against the NCBI BLAST nucleotide collection database using the program Megablast (The Pennsylvania State University, University Park, TX, USA) with default settings.

## 3. Results

### 3.1. Microsatellite Analysis

#### 3.1.1. Genetic Diversity and Population Size

There was no evidence for large allelic dropout, and the presence of null alleles was assumed in loci Hmo 13, 33, 36, 37, 39, and 40 in Balaton; Hmo 02, 13, 33, and 39 in Tisza; locus Hmo 36 in silver carp; and loci 33 and 37 in bighead carp stocks because of the general excess of homozygotes. The following loci showed deviation from the HW equilibrium: all loci in the Balaton stock; Hmo33 in the stock of inflow in Balaton; Hmo 01, 02, 13, 33, and 39 in the Tisza stock; Hmo 34, 36, and 40 in the native silver carp population; and Hmo 02, 13, 33, 37, 39, and 40 in the native bighead carp population.

Regarding the diversity data in the two studied stocks, the Balaton stock showed significantly higher values for the number of alleles (Na) and the effective number of alleles (Neff). Allelic richness was also higher in the Balaton stock, but the difference was not significant. In the case of private allelic richness (ARp), the Tisza stock showed a significantly higher value. Heterozygosity values were similar in all stocks/populations. The diversity of individuals collected in the inflow area of Lake Balaton was lower compared with both the Balaton stock and the Tisza stock; however, a significant difference could only be detected in the Na compared with Balaton and in ARp compared with the Tisza.

The gene bank stocks of both species showed significant discrepancies from the Balaton stock in the case of NA values and from the bighead stock in the case of Ne values. The ARp showed a difference between the Balaton inflow and bighead stocks (Table 1).

The values of the stock sizes (Ne) in the three Hungarian stocks estimated by the linkage disequilibrium method were 49.7 (CI 95%: 45.5–54.6), 129.0 (CI 95%: 9.9-infinite), and 98.4 (CI 95%: 50.1–570.4) individuals in the Balaton, Balaton inflow, and Tisza stocks, respectively. Unexpectedly, the Wilcoxon signed-rank test for heterozygosity excess performed by BOTTLENECK did not support a recent population bottleneck in any of these stocks.

#### 3.1.2. Genetic Structure

The global F_st_ was 0.142 (95% CI: 0.105–0.187) and 0.139 (95% CI: 0.100–0.186) without and with ENA correction, indicating only moderate genetic distances. The pairwise F_st_ and Cavalli-Sforza and Edwards genetic distance values between stock pairs are shown in Table 2 and Table 3 (with corrections on null alleles). The natural populations were more genetically close to the silver carp, and the largest distance was found between the Tisza and gene bank bighead carp stocks. Moreover, the Tisza stock also showed a significant separation from the Lake Balaton stock and its inflow stocks. The genetic distance between the Balaton, Balaton inflow, and gene bank silver carp stocks was minimal.

**Figure 2:**
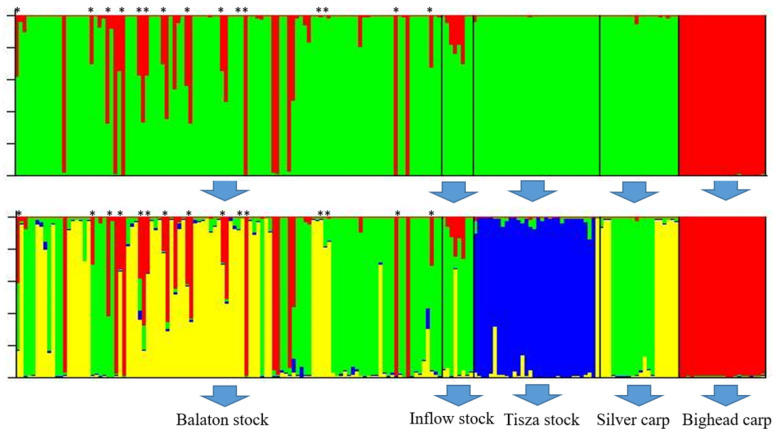
Hierarchical structure of the five stocks/populations based on the microsatellite data. The hybrid individuals based on the mitochondrial DNA analysis are marked with an asterisk.

Hierarchical clustering by STRUCTURE software (Figure 2, Table S4) resulted in the most probable K = 2 clusters in the first step. The two clusters separated the bighead carp population and the species hybrid individuals from the silver carp individuals. The Tisza population did not contain hybrids. Clustering the three Hungarian stocks and silver carp live gene bank stock resulted in the K = 4 cluster number. In this case, the first cluster separated the hybrid individuals (red) with a larger portion of the bighead carp genome, and the second cluster contained the individuals in the Tisza stock (blue). The remaining two clusters were found in both the Hungarian stocks in the Balaton region and silver carp live gene bank stocks (yellow and green). Although two genetic clusters were separated, the two sampling places of Balaton and its inflow could not be separated based on the remaining genetic clusters.

### 3.2. Mitochondrial DNA Analysis

**Figure 3:**
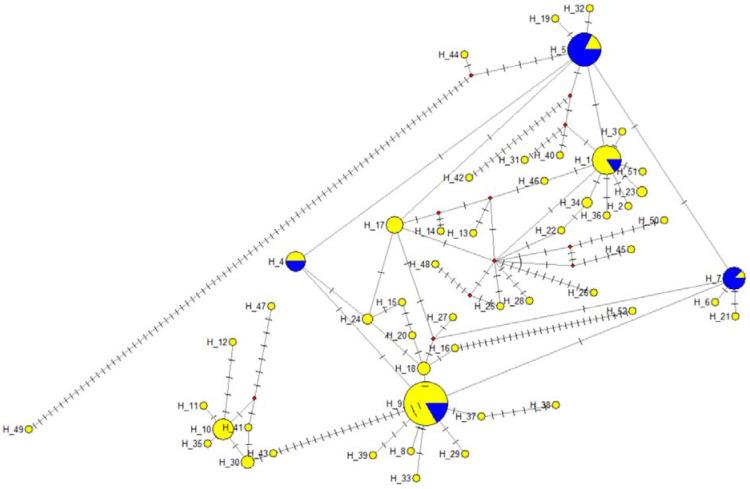
The mtDNA haplotype networks for COI mitochondrial DNA sequences. Yellow: Balaton stock, blue: Tisza stock. The size of the circles represents the number of haplotypes.

In total, 138 mtDNA sequences were analyzed, resulting in 52 haplotypes (Figure 3).

Within the haplotype groups, the number of polymorph sites was 175 (Figure S1), and 43 sites were parsimony informative. The diversity data of the COI gene sequences are presented in Table 4. Blasting of the haplotypes identified eight as bighead carp sequences, and the remaining belonged to the silver carp species. Blasting the sequences resulted in 100% identity in five of the SC haplotypes (hap1-MF122391.1, hap4-MF122388.1, hap5-MF122397.1, hap7-MF122392.1, and hap9-MF122390.1) and one of the BHC haplotypes (hap10-MF122412.1). All the haplotypes identified in the NCBI nucleotide collection database had an origin of the Yangtze River. The Balaton stock contained all the haplotypes, from which hap1, hap4, hap5, hap7, and hap9 were dominant in the silver carp species. In the case of the bighead carp, the h10 was dominant. In the Tisza stock, only five haplotypes were identified, belonging to the hap1, hap4, hap5, hap 7, and hap9 dominant haplotypes.

Based on the STRUCTURE analysis of the microsatellite data, 31 individuals were clustered as hybrids, of which 25 individuals were confirmed by the allele length of the Hmo1 and Hmo3 microsatellites that have different allele size ranges in the bighead carp and silver carp [40]. The mtDNA sequences confirmed 15 individuals from the 31 hybrids based on STRUCTURE analysis, of which 4 individuals did not show the bighead carp microsatellite allele variants. Moreover, one individual was assigned as a hybrid based on neither STRUCTURE nor MS allele variants. All the hybrid individuals (independently from the detection method) belonged to the Balaton stock.

## 4. Discussion

Bigheaded carps show different genetic and stock/population characteristics in their native range and in their introduced habitats. The loss of genetic diversity results in population decline in the native habitat (due to the degradation of growth, adaptability, and fecundity), but in the Mississippi River Basin, the two introduced species became invasive parallel with the ability of hybridization in natural waters [20]. The genetic diversity of the species based on microsatellite data in this study (AR ranging between 4.6 and 6.4) was similar to the literature data. Farrington et al. [21] measured allelic richness of 4.7 in North American populations, which was lower than that in its native habitat (AR = 5.7–6.5) or that determined by [41] in the same area (AR = 6.1). The average heterozygosity in the USA (0.66- [21] or 0.60- [41]) was also similar to the heterozygosity in our “natural” populations/stocks (0.61–0.78). Lu et al. [20] provided a detailed review of previous mitochondrial DNA diversity patterns in native and introduced habitats. In contrast to microsatellite studies, previous data on the Danube River area are available for mitochondrial DNA (where sample collection was partially in the Tisza River (Szeged location) [22]). Although haplotype diversity and nucleotide diversity were lower in the Danube region than in the native habitats (Yangtze, Pearl, Amur), they were found to be higher than those in North American stocks. The Balaton stock presented the same value as the Danube stock for haplotype diversity (HD: 0.85 vs. 0.84) but reported higher values for the nucleotide diversity (ND: 0.005 vs. 0.018). In the case of the Tisza population, these values proved to be lower and show similarities with the values of the North American stock (Mississippi River Basin) (HD: 0.73 vs.0.73 and ND: 0.002 vs. 0.003) [20].

Thus, the genetic diversity of silver carp stocks in the two stocks is the same or slightly lower than that of native populations. Silver carp also show similar or higher diversity compared with other invasive species that also occur in the region. The pumpkinseed sunfish (*Lepomis gibbosus*) was introduced at the end of the 19th century in several countries in Europe. The species has spread from Germany to Central Europe through the Danube River [42]. This Mid-European linage includes populations with high and low genetic diversity (the heterozygosity ranges between 0.31–0.75 and mean allelic richness between 2 and 5). Another invasive species, the topmouth gudgeon (*Pseudorasbora parva*) was introduced to Europe along with the bigheaded carp [43]. The populations near the introduction site (e.g., Hungary) show a higher level of diversity and differentiation. The species follows a stepping stone colonization pattern (with the lowest diversity in the recently established UK populations). However, the Hungarian stocks have low allelic richness (2.90–3.05) and heterozygosity (0.52–0.66).

The high diversity of the Hungarian bigheaded carp stocks could be rooted in their origin. The main haplotypes in both stocks correspond to the Yangtze River. This confirms the data in the review by [20], where the main gene flow to the Danube stock was from the Yangtze and partially from the Amur River. Bigheaded carps were first introduced to Hungary in 1963 from China (originated from natural reproduction). This introduction was followed by several more cases in the years 1963–1967 when artificially propagated stocks were introduced from the Soviet Union. [44] The Yangtze represented the highest diversity from the native populations. Repeated aquaculture introductions from these source populations could result in higher diversity.

In the Balaton stock, 40–45% of the maternally inherited mitochondrial sequences were derived from bighead carp in the hybrid individuals, classified by the species-specific microsatellites and the clustering by structure. Bighead carp females are only willing to spawn in a strong flood, while in the case of silver carp, this is possible even at moderately increasing water levels [13]. However, the male bighead carp is capable of sperm production, so during hybridization, the *H. molitrix* female x *H. nobilis* male combination is more probable under natural conditions [45]. Thus, the predominance of *H. molitrix* haplotypes among the hybrid individuals in Lake Balaton was expected, in contrast to the observed 45%. In the case of hormonally induced propagation under aquaculture conditions, the water level increase is not a limiting factor of reproduction [46]. In addition, the production of hybrid bigheaded carps is part of the aquaculture practice [47,48]; therefore, the proportion of bighead carp haplotypes may increase. In addition, the estimated level of the hybrids may have been underestimated based on the number of used markers. The hybrids are fertile and can be crossed with both parental species, and this is part of the aquaculture practice. The efficiency of the hybrid identification in backcrossed, F2, and later generations are strongly influenced by random segregation and recombination of the chromosomes. The mathematical modeling of hybrid identification indicates the necessity of upwards of 70 markers for reliable discrimination (depending on the number of generations) between the pure species and later hybrid generations [49].

Based on the microsatellite data, all ten loci in the Lake Balaton stock showed a significant deviation from the HW equilibrium. However, no bottleneck effect was observed, and this stock showed the highest diversity with microsatellite markers. We also obtained a haplotype number similar to the native habitat [22]. Thus, we cannot assume the presence of a reproductive stock (i.e., no population formed) in the lake, but suggest that the current genetic structure developed as a result of repeated introductions and hybridization (both intended stockings and unintended escapes from fish ponds [19]). This is also confirmed by the fact that three genetic clusters can be observed in the lake, which cannot be distinguished by age, gender, or localization. It is assumed that the juveniles entering the lake came from several sources (fish farms), although this could not be confirmed because of the small sample size.

The Tisza stock showed a different genetic pattern. Based on the mtDNA data, the stock consists of pure silver carp individuals, and hybrids were not detected. The microsatellite diversity and the haplotype number were similar to the Mississippi population in North America [21]. The lack of the bottleneck effect was unexpected since significant fish mortality was observed in the habitat in 2000 due to cyanide pollution affecting the river [10]. Surviving and locally reproducing stocks appear to have achieved relatively high diversity in a short period of time, supplemented by individuals that escaped from fish farms during floods. The stock found in this river habitat appears to have significant reproductive potential (i.e., could form a valid population) and may become invasive given environmental factors changing because of climate change (e.g., more extended feeding period, extreme rainfall distribution).

The Danube River is a part of the Southern invasion corridor in Europe and can be considered an “invasion gateway” [50]. Several invasive fish species (e.g., bighead goby, *Neogobius kessleri*) have been introduced and settled in Hungary via the Danube River and extended further upstream [51]. The topmouth gudgeon (*Pseudorasbora parva*), which settled simultaneously with bigheaded carps, has now become an invasive species in Europe [43]. The spread of bigheaded carps within the corridor was probably hindered by the lack of self-sustaining stocks, which may have been strongly influenced by the suboptimal environmental factors (flooding, temperature) affecting the reproduction of the species. However, recent investigations demonstrated that reproduction could occur in bigheaded carp species in Western Europe [7]. The reproduction of silver carp in the Tisza River has already been demonstrated and is showing an accelerating trend over the last 20 years [12]. In the Tisza tributary Körös River, the species was not yet detectable in a 2009 survey, while in 2019 it had the second-highest abundance (24.1%), and its mass reproduction was also confirmed [52]. According to the model of [53], successful removal of bigheaded carp stocks requires continuous fishing of all age classes. Commercial fishing for the adult age class was maintained in the Tisza River until 2013, but changes in the legislation (Act No. CII of 2013 concerning fisheries and the protection of fish) then abolished commercial fishing, and ineffective angling became the exclusive mode of fish removal [52]. Predictably, this type of management coupled with the increasing water temperature could not control the gradation of the bigheaded carp species.

## 5. Conclusions

Our investigation highlights that depending on the habitat quality, the reproduction of the silver carp varies in riverine habitats. The genetic information suggests that the species may become invasive similarly in the North American populations. It is important to develop detailed management plans and monitoring activities to control the spread of the silver carp. It seems that the distinct Balaton stock does not pose a significant hazard because of the lack of regular reproduction. The elimination of the stock could be implemented by the rigorous monitoring of the inflow waters and the continuous selective fishing of the adult individuals. However, the Tisza River represents a different situation, where management coupled with the effects of climate change could not control the gradation of the bigheaded carp species.

## Figures and Tables

**Table 1:** Genetic diversity data of stocks/populations studied.

	Balaton	Inflow of Balaton	Tisza	Silver Carp	Bighead Carp
Na	14.00 ± 4.81 ^a^	4.70 ± 0.94 ^b^	8.00 ± 4.18 ^b^	7.70 ± 3.40 ^b^	6.20 ± 1.81 ^b^
Neff	5.64 ± 2.91 ^b^	3.18 ± 0.83 ^ab^	4.72 ± 2.71 ^ab^	4.61 ± 2.05 ^ab^	2.97 ± 0.91 ^a^
Ho	0.68 ± 0.14	0.78 ± 0.22	0.61 ± 0.18	0.74 ± 0.15	0.58 ± 0.26
uHe	0.77 ± 0.11	0.70 ± 0.12	0.72 ± 0.17	0.75 ± 0.12	0.64 ± 0.12
F	0.10 ± 0.19	−0.17 ± 0.27	0.11 ± 0.17	0.02 ± 0.20	0.07 ± 0.37
AR	6.46 ± 1.88	4.60 ± 0.84	5.53 ± 2.30	5.67 ± 2.02	5.52 ± 1.10
AR_p_	0.70 ± 0.45 ^ab^	0.37 ± 0.48 ^a^	2.05 ± 1.45 ^c^	1.15 ± 0.80 ^abc^	1.90 ± 1.32 ^bc^

Na: number of alleles, Neff: effective number of alleles, uHe: unbiased expected heterozygosity, Ho: observed heterozygosity values, F: inbreeding coefficient, AR: allelic richness, AR_p_: private allelic richness. If indicated, ‘^a^’, ‘^b^’ and ‘^c^’ upper case letters indicate significant (*p* < 0.05) differences between the groups.

**Table 2:** Pairwise F_st_ (below diagonal) and Cavalli-Sforza and Edwards genetic distance (above diagonal). Bootstrap 95% intervals are shown in Table S3.

F_st_/uNeiD	Balaton	Inflow of Balaton	Silver Carp	Bighead Carp	Tisza
Balaton		0.378	0.341	0.639	0.519
Inflow of Balaton	0.015		0.445	0.717	0.610
Silver carp	0.015	0.039		0.806	0.542
Bighead carp	0.213	0.258	0.279		0.831
Tisza	0.130	0.169	0.138	0.305	

**Table 3:** Pairwise F_st_ using the ENA correction (below diagonal) and Cavalli-Sforza and Edwards genetic distance using the INA correction (above diagonal). Bootstrap 95% intervals are shown in Table S3.

F_st_/uNeiD	Balaton	Inflow of Balaton	Silver Carp	Bighead Carp	Tisza
Balaton		0.387	0.340	0.640	0.523
Inflow of Balaton	0.021		0.447	0.714	0.624
Silver carp	0.015	0.040		0.805	0.555
Bighead carp	0.207	0.243	0.265		0.822
Tisza	0.129	0.173	0.139	0.290	

**Table 4:** Diversity data of the mtDNA sequences in the two natural populations.

Stock	S	Eta	Hd (Mean ± SD)	Pi (Mean ± SD)
Balaton	175	211	0.931 ± 0.00029	0.02256 ± 0.0000116
Tisza	4	4	0.736 ± 0.059	0.0027 ± 0.00027

S—Number of polymorphic (segregating) sites, Eta—total number of mutations, Hd—haplotype diversity, Pi—nucleotide diversity, SD-Standard Deviation.

## Data Availability

All data supporting the reported results can be found as Supplementary Material (Tables S1 and S2).

## Supplementary Materials

The following are available online at https://www.mdpi.com/article/10.3390/ani11072018/s1, Table S1: The raw data of the experiment. Table S2. The sequences of the mtDNA haplotypes. Table S3: Bootstrap 95% intervals for F_st_ and Cavalli-Sforza and Edwards genetic distance. Table S4: The mean log likelihood data and Evanno’s delta K of the hierarchical STRUCTURE analysis. Figure S1: Polymorphic sites in the haplotypes.
