# Supplementary material for: Comparison of the Genetic Structure of Invasive Bigheaded Carp (Hypophthalmichthys spp.) Populations in Central-European Lacustrine and Riverine Habitats
Source: Animals (Basel). 2021 Jul 6;11(7):2018. doi: 10.3390/ani11072018 (PMC8300242; doi:10.3390/ani11072018)
Supplement: Supplementary file 1 [file animals-11-02018-s001.zip › Supplementary material/Figure S1.pdf]

Figure S1.
Polymorph sites in the haplotypes
Title:Comparison of the genetic structure of invasive bigheaded carp (Hypophthalmichthys spp.) populations in Central-European lacustrine and riverine habitats
Authors:Tamás Molnár*, István Lehoczky*, Erika Edviné Meleg , Gergely Boros, András Specziár, Attila Mozsár, Zoltán Vitál, Vilmos Józsa, Wahiba Allele, Béla Urbányi and Balázs Kovács

```
MATRIX
[                10        20        30        40        50        60        70        80        90        100       110       120       130       140       150       160       170      ]
[                 *         *         *         *         *         *         *         *         *         *         *         *         *         *         *         *         *      ]
Hap_1    GTGGTTTGTACGCTAAGGAGGTCTGAGAAGCACGGGTTTGAAGGATCTGTATATACCGTAGAAGTGGGACTGAGGCAGTTATACTCCAAACGGGCGTGTGGTTGTCATTACTGCAGGTGTGTAACTCTTGGGCATAACAATGGTAACAGGTCGTTGAGTCTTAGAGCGGCGTTCC
Hap_2    ............................................................................................................................................................................C..
Hap_3    ...............................................A...............................................................................................................................
Hap_4    .................................................................................................................T............T................................................
Hap_5    ..............................................................................................................................T................................................
Hap_6    ....................................................G.....A..........................................................C........T................................................
Hap_7    ....................................................G................................................................C........T................................................
Hap_8    ....................................................G............................................................T...C........T.............A..................................
Hap_9    ....................................................G............................................................T...C........T................................................
Hap_10   ...............G.......CA................G.A......G.GC.T.A.G.G.........A..A....CG......G.............GC..........T..TC......T.T.C..........G.....G.............................
Hap_11   ...............G.......CA................G.A......GAGC.T.A.G.G.........A..A....CG......G.............GC..........T..TC......T.T.C..........G.....G.............................
Hap_12   .....AATC......G.......CA................G.A......G.GC.T.A.G.G.........A..A....CG......G.............GC..........T..TC......T.T.C..........G.....G.............................
Hap_13   ....AA..A.A....................................................................................................................................................................
Hap_14   .....A..A........C.C..........................................................................................................T................................................
Hap_15   .....AA........................................................................................................T............T................................................
Hap_16   .....A..A...........................................G............................................................T...C........T................................................
Hap_17   .....A........................................................................................................................T................................................
Hap_18   .....A..............................................G............................................................T...C........T................................................
Hap_19   ......G.......................................................................................................................T................................................
Hap_20   .....AA.............................................G............................................................T...C........T................................................
Hap_21   A.....................G.............................G................................................................C........T................................................
Hap_22   .....AA..........................................................................................................................................................................
Hap_23   ..A............................................................................................................................................................................
Hap_24   .....A.........................................................................................................T............T................................................
Hap_25   .....A.........................................................................................................................................................C...T.......
Hap_26   .....A..........C...AA......................CC....................................................C.......................C.....C............................CCC................
Hap_27   ...GA...............................................G................................................................C........T................................................
Hap_28   .....A...............................................................................................................................................CCC............C......
Hap_29   A...............C.C.................................G............................................................T...C........T................................................
Hap_30   .......................CA................G.A......G.GC.T.A.G.G.........A..A....CG......G.............GC..........T..TC......T.T.C..........G.....G.............................
```

```
MATRIX
[                 10        20        30        40        50        60        70        80        90       100       110       120       130       140       150       160       170      ]
[                  *         *         *         *         *         *         *         *         *         *         *         *         *         *         *         *         *       ]
Hap_31    ...................A.............................................................................C....................AG.............................C........C...T............
Hap_32    ................T.............................................................................................................T.....................................G..........
Hap_33    ....................................................G............................................................T...C........T.........................CG.....................
Hap_34    ......A........................................................................................................................................................................
Hap_35    ...............G.......CA................G.A......G.GC.T.A.G.G.........A..A....CG......G.............GC..........T..TC......T.T.C..........G.....G..........................C..
Hap_36    .....................................................................................................................................TG........................................
Hap_37    ....................................................G............................................................T...C........T......................................A.........
Hap_38    ....................................................G............................................................T...C..T.....T.........C............T........G......A...GC....
Hap_39    ...........................C........CCC.............G............................................................T...C........T................................................
Hap_40    .............................................C.................................................................G......A........................................................
Hap_41    .......................CA................G.A......G.GC.T.A.G.G.........A..A....CG......G.............GC..........T..TC......T.T.C..........G.....G...................A.........
Hap_42    ..................................................................C.........................TA...A.AA..A...........A..A.......T..AA.................T...C....A.....A.A.A.....T.
Hap_43    .......................CA................G.A......G.GC.T.A.G.G.........A..A....CG......G.............GC..........T...C......T.T.C..........G.....G.............................
Hap_44    ..T.........G.................................G................................G.G..G.........................................T..............................A.................
Hap_45    ....A......................................................................................................................................C.......CAA.......C...G.............
Hap_46    ........A......................................................................................................................................................................
Hap_47    ....CA..AT.....G.......CA................G.A......G.GC.T.A.G.G.........A..A....CG......G.............GC..........T..TC......TGT.C..........G.....G...................AG........
Hap_48    ....A..............C..................................C..........................................................................................C.................A...A.GC....
Hap_49    .G......GT.TG.G....AA.G..GAGC.GTAAA.G....G.A.AG..G..G...G...A.GAGA.TGGG.TA.GTAAG.GCTGGGCTCGA..GAGA.A..GA.TC.GGGG.TCA.C....C...T....AG..G.ACT.T.G........C..T.A.GC..A..GA.....TT
Hap_50    ....A..............A......................T........................................................................A.......................T..T.T.A..T.TC......................
Hap_51    ..A............................................................................................................................................................................
Hap_52    ....A...A....C.......G..C.C..C.....C...TTTT..C..CC..G...................................................C..AG..GCT...C.....C..T....C...........C....T.C......T....G....A..CC...
```
