# Supplementary material for: Comparison of the Genetic Structure of Invasive Bigheaded Carp (Hypophthalmichthys spp.) Populations in Central-European Lacustrine and Riverine Habitats
Source: Animals (Basel). 2021 Jul 6;11(7):2018. doi: 10.3390/ani11072018 (PMC8300242; doi:10.3390/ani11072018)
Supplement: Supplementary file 1 [file animals-11-02018-s001.zip › Supplementary material/Table S2.pdf]

Table S2

The sequences of the 52 haplotypes.

Title: Comparison of the genetic structure of invasive bigheaded carp (Hypophthalmichthys spp.) populations in Central-European lacustrine and riverine habitats

Authors: Tamás Molnár *, István Lehoczky*, Erika Edviné Meleg, Gergely Boros, András Specziár, Attila Mozsár, Zoltán Vitál, Vilmos Józsa, Wahiba Allele, Béla Urbányi and Balázs Kovács

>hap1

CGGTCTGTAAGGAGTATTGTAATTCCAGCAGCTAAAACTGGTAAGGATAAGAGAAGAAGTACGGCTGTTACG
AGCACAGCTCAAACAAAGAGAGGTGTTTGATATTGAGAGATGGCTGGTGGTTTTATATTAATAGTTGTGGTGA
TAAAGTTAATTGCTCCTAAAATTGATGACACACCTGCTAGGTGAAGAGAGAAAATTGTTAGGTCTACGGATGC
TCCTGCGTGGGCAAGATTGCCCGCGAGTGGTGGATAAACTGTTCATCCTGTTCCGGCCCCGGCCTCAACACCA
GAAGAGGCTAGTAGTAGAAGGAAAGATGGGGGTAGGAGTCAAAAGCTTATATTATTTATTCGTGGGAATGCT
ATATCAGGTGCCCCAATCATTAGGGGTACGAGTCAGTTTCCAAACCCTCCAATAAGGATCGGTATTACTATAAA
GAAAATTATTACGAAGGCATGGGCAGTAACAATAACATTATAAATTTGGTCATCACCCAGAAGTGATCCGGGT
TGGCTTAGTTCGGCTCGAATGAGAAGGCTTAGGGCGGTTCCC

> hap2

CGGTCTGTAAGGAGTATTGTAATTCCAGCAGCTAAAACTGGTAAGGATAAGAGAAGAAGTACGGCTGTTACG
AGCACAGCTCAAACAAAGAGAGGTGTTTGATATTGAGAGATGGCTGGTGGTTTTATATTAATAGTTGTGGTGA
TAAAGTTAATTGCTCCTAAAATTGATGACACACCTGCTAGGTGAAGAGAGAAAATTGTTAGGTCTACGGATGC
TCCTGCGTGGGCAAGATTGCCCGCGAGTGGTGGATAAACTGTTCATCCTGTTCCGGCCCCGGCCTCAACACCA
GAAGAGGCTAGTAGTAGAAGGAAAGATGGGGGTAGGAGTCAAAAGCTTATATTATTTATTCGTGGGAATGCT
ATATCAGGTGCCCCAATCATTAGGGGTACGAGTCAGTTTCCAAACCCTCCAATAAGGATCGGTATTACTATAAA
GAAAATTATTACGAAGGCATGGGCAGTAACAATAACATTATAAATTTGGTCATCACCCAGAAGTGATCCGGGT
TGGCTTAGTTCGGCTCGAATGAGAAGGCTTAGGGCGGTCCCC

> hap3

CGGTCTGTAAGGAGTATTGTAATTCCAGCAGCTAAAACTGGTAAGGATAAGAGAAGAAGTACGGCTGTTACG
AGCACAGCTCAAACAAAGAGAGGTGTTTGATATTGAGAGATGGCAGGTGGTTTTATATTAATAGTTGTGGTGA
TAAAGTTAATTGCTCCTAAAATTGATGACACACCTGCTAGGTGAAGAGAGAAAATTGTTAGGTCTACGGATGC
TCCTGCGTGGGCAAGATTGCCCGCGAGTGGTGGATAAACTGTTCATCCTGTTCCGGCCCCGGCCTCAACACCA
GAAGAGGCTAGTAGTAGAAGGAAAGATGGGGGTAGGAGTCAAAAGCTTATATTATTTATTCGTGGGAATGCT
ATATCAGGTGCCCCAATCATTAGGGGTACGAGTCAGTTTCCAAACCCTCCAATAAGGATCGGTATTACTATAAA
GAAAATTATTACGAAGGCATGGGCAGTAACAATAACATTATAAATTTGGTCATCACCCAGAAGTGATCCGGGT
TGGCTTAGTTCGGCTCGAATGAGAAGGCTTAGGGCGGTTCCC

> hap4

CGGTCTGTAAGGAGTATTGTAATTCCAGCAGCTAAAACTGGTAAGGATAAGAGAAGAAGTACGGCTGTTACG
AGCACAGCTCAAACAAAGAGAGGTGTTTGATATTGAGAGATGGCTGGTGGTTTTATATTAATAGTTGTGGTGA
TAAAGTTAATTGCTCCTAAAATTGATGACACACCTGCTAGGTGAAGAGAGAAAATTGTTAGGTCTACGGATGC
TCCTGCGTGGGCAAGATTGCCCGCGAGTGGTGGATAAACTGTTCATCCTGTTCCGGCCCCGGCCTCAACACCA
GAAGAGGCTAGTAGTAGAAGGAAAGATGGGGGTAGGAGTCAAAAGCTTATATTATTTATTCGTGGGAATGCT
ATATCAGGTGCTCCAATCATTAGGGGTACGAGTCAGTTTCCAAACCCTCCAATAAGGATTGGTATTACTATAAA

GAAAATTATTACGAAGGCATGGGCAGTAACAATAACATTATAAATTTGGTCATCACCCAGAAGTGATCCGGGT
TGGCTTAGTTCGGCTCGAATGAGAAGGCTTAGGGCGGTTCCC

> hap5

CGGTCTGTAAGGAGTATTGTAATTCCAGCAGCTAAAACTGGTAAGGATAAGAGAAGAAGTACGGCTGTTACG
AGCACAGCTCAAACAAAGAGAGGTGTTTGATATTGAGAGATGGCTGGTGGTTTTATATTAATAGTTGTGGTGA
TAAAGTTAATTGCTCCTAAAATTGATGACACACCTGCTAGGTGAAGAGAGAAAATTGTTAGGTCTACGGATGC
TCCTGCGTGGGCAAGATTGCCCGCGAGTGGTGGATAAACTGTTCATCCTGTTCCGGCCCCGGCCTCAACACCA
GAAGAGGCTAGTAGTAGAAGGAAAGATGGGGGTAGGAGTCAAAAGCTTATATTATTTATTCGTGGGAATGCT
ATATCAGGTGCCCCAATCATTAGGGGTACGAGTCAGTTTCCAAACCCTCCAATAAGGATTGGTATTACTATAAA
GAAAATTATTACGAAGGCATGGGCAGTAACAATAACATTATAAATTTGGTCATCACCCAGAAGTGATCCGGGT
TGGCTTAGTTCGGCTCGAATGAGAAGGCTTAGGGCGGTTCCC

> hap6

CGGTCTGTAAGGAGTATTGTAATTCCAGCAGCTAAAACTGGTAAGGATAAGAGAAGAAGTACGGCTGTTACG
AGCACAGCTCAAACAAAGAGAGGTGTTTGATATTGAGAGATGGCTGGTGGTTTTATATTAATAGTTGTGGTGA
TGAAGTTAATTGCTCCTAAAATTGATGACACACCTGCTAGGAGAAGAGAGAAAATTGTTAGGTCTACGGATGC
TCCTGCGTGGGCAAGATTGCCCGCGAGTGGTGGATAAACTGTTCATCCTGTTCCGGCCCCGGCCTCAACACCA
GAAGAGGCTAGTAGTAGAAGGAAAGATGGGGGTAGGAGTCAAAAGCTTATATTATTTATTCGTGGGAATGCT
ATATCAGGTGCCCCAATCATTAGGGGCACGAGTCAGTTTCCAAACCCTCCAATAAGGATTGGTATTACTATAAA
GAAAATTATTACGAAGGCATGGGCAGTAACAATAACATTATAAATTTGGTCATCACCCAGAAGTGATCCGGGT
TGGCTTAGTTCGGCTCGAATGAGAAGGCTTAGGGCGGTTCCC

> hap7

CGGTCTGTAAGGAGTATTGTAATTCCAGCAGCTAAAACTGGTAAGGATAAGAGAAGAAGTACGGCTGTTACG
AGCACAGCTCAAACAAAGAGAGGTGTTTGATATTGAGAGATGGCTGGTGGTTTTATATTAATAGTTGTGGTGA
TGAAGTTAATTGCTCCTAAAATTGATGACACACCTGCTAGGTGAAGAGAGAAAATTGTTAGGTCTACGGATGC
TCCTGCGTGGGCAAGATTGCCCGCGAGTGGTGGATAAACTGTTCATCCTGTTCCGGCCCCGGCCTCAACACCA
GAAGAGGCTAGTAGTAGAAGGAAAGATGGGGGTAGGAGTCAAAAGCTTATATTATTTATTCGTGGGAATGCT
ATATCAGGTGCCCCAATCATTAGGGGCACGAGTCAGTTTCCAAACCCTCCAATAAGGATTGGTATTACTATAAA
GAAAATTATTACGAAGGCATGGGCAGTAACAATAACATTATAAATTTGGTCATCACCCAGAAGTGATCCGGGT
TGGCTTAGTTCGGCTCGAATGAGAAGGCTTAGGGCGGTTCCC

> hap8

CGGTCTGTAAGGAGTATTGTAATTCCAGCAGCTAAAACTGGTAAGGATAAGAGAAGAAGTACGGCTGTTACG
AGCACAGCTCAAACAAAGAGAGGTGTTTGATATTGAGAGATGGCTGGTGGTTTTATATTAATAGTTGTGGTGA
TGAAGTTAATTGCTCCTAAAATTGATGACACACCTGCTAGGTGAAGAGAGAAAATTGTTAGGTCTACGGATGC
TCCTGCGTGGGCAAGATTGCCCGCGAGTGGTGGATAAACTGTTCATCCTGTTCCGGCCCCGGCCTCAACACCA
GAAGAGGCTAGTAGTAGAAGGAAAGATGGGGGTAGGAGTCAAAAGCTTATATTATTTATTCGTGGGAATGCT
ATATCAGGTGCTCCAATCATTAGGGGCACGAGTCAGTTTCCAAACCCTCCAATAAGGATTGGTATTACTATAAA
GAAAATTATTACGAAGGCATGGGCAGTAACAATAACATTAAAAATTTGGTCATCACCCAGAAGTGATCCGGGT
TGGCTTAGTTCGGCTCGAATGAGAAGGCTTAGGGCGGTTCCC

> hap9

CGGTCTGTAAGGAGTATTGTAATTCCAGCAGCTAAAACTGGTAAGGATAAGAGAAGAAGTACGGCTGTTACG
AGCACAGCTCAAACAAAGAGAGGTGTTTGATATTGAGAGATGGCTGGTGGTTTTATATTAATAGTTGTGGTGA
TGAAGTTAATTGCTCCTAAAATTGATGACACACCTGCTAGGTGAAGAGAGAAAATTGTTAGGTCTACGGATGC

TCCTGCGTGGGCAAGATTGCCCGCGAGTGGTGGATAAACTGTTCATCCTGTTCCGGCCCCGGCCTCAACACCA
GAAGAGGCTAGTAGTAGAAGGAAAGATGGGGGTAGGAGTCAAAAGCTTATATTATTTATTCGTGGGAATGCT
ATATCAGGTGCTCCAATCATTAGGGGCACGAGTCAGTTTCCAAACCCTCCAATAAGGATTGGTATTACTATAAA
GAAAATTATTACGAAGGCATGGGCAGTAACAATAACATTATAAATTTGGTCATCACCCAGAAGTGATCCGGGT
TGGCTTAGTTCGGCTCGAATGAGAAGGCTTAGGGCGGTTCCC

> hap10

CGGTCTGTAAGGAGTATTGTAATTCCAGCAGCTAAAACTGGTAAGGATAGGAGAAGAAGTACGGCCGTTACA
AGCACAGCTCAAACAAAGAGAGGTGTTTGATATTGGGAAATGGCTGGTGGTTTTATGTTAATAGTTGTGGTGA
TGAAGTTAATTGCCCCTAAAATTGATGATACACCTGCTAAGTGGAGGGAGAAAATTGTTAGGTCTACGGATGC
TCCTGCGTGAGCAAGATTACCCGCGAGTGGCGGGTAAACTGTTCATCCTGTTCCGGCCCCGGCCTCGACACCA
GAAGAGGCTAGTAGTAGAAGGAAAGAGGGGGGCAGGAGTCAAAAGCTTATATTATTTATTCGTGGGAATGCT
ATATCAGGTGCTCCAATCATTAGTGGCACGAGTCAGTTTCCAAATCCTCCAATAAGGATTGGTATCACTATAAA
GAAAATTATTACGAAGGCATGGGCAGTAACAATAACGTTATAAATTTGGTCATCGCCCAGAAGTGATCCGGGT
TGGCTTAGTTCGGCTCGAATGAGAAGGCTTAGGGCGGTTCCC

> hap11

CGGTCTGTAAGGAGTATTGTAATTCCAGCAGCTAAAACTGGTAAGGATAGGAGAAGAAGTACGGCCGTTACA
AGCACAGCTCAAACAAAGAGAGGTGTTTGATATTGGGAAATGGCTGGTGGTTTTATGTTAATAGTTGTGGTGA
AGAAGTTAATTGCCCCTAAAATTGATGATACACCTGCTAAGTGGAGGGAGAAAATTGTTAGGTCTACGGATGC
TCCTGCGTGAGCAAGATTACCCGCGAGTGGCGGGTAAACTGTTCATCCTGTTCCGGCCCCGGCCTCGACACCA
GAAGAGGCTAGTAGTAGAAGGAAAGAGGGGGGCAGGAGTCAAAAGCTTATATTATTTATTCGTGGGAATGCT
ATATCAGGTGCTCCAATCATTAGTGGCACGAGTCAGTTTCCAAATCCTCCAATAAGGATTGGTATCACTATAAA
GAAAATTATTACGAAGGCATGGGCAGTAACAATAACGTTATAAATTTGGTCATCGCCCAGAAGTGATCCGGGT
TGGCTTAGTTCGGCTCGAATGAGAAGGCTTAGGGCGGTTCCC

> hap12

CGGTCTGTAAGGAGTAAATCAATTCCAGCAGCTAAAACTGGTAAGGATAGGAGAAGAAGTACGGCCGTTACA
AGCACAGCTCAAACAAAGAGAGGTGTTTGATATTGGGAAATGGCTGGTGGTTTTATGTTAATAGTTGTGGTGA
TGAAGTTAATTGCCCCTAAAATTGATGATACACCTGCTAAGTGGAGGGAGAAAATTGTTAGGTCTACGGATGC
TCCTGCGTGAGCAAGATTACCCGCGAGTGGCGGGTAAACTGTTCATCCTGTTCCGGCCCCGGCCTCGACACCA
GAAGAGGCTAGTAGTAGAAGGAAAGAGGGGGGCAGGAGTCAAAAGCTTATATTATTTATTCGTGGGAATGCT
ATATCAGGTGCTCCAATCATTAGTGGCACGAGTCAGTTTCCAAATCCTCCAATAAGGATTGGTATCACTATAAA
GAAAATTATTACGAAGGCATGGGCAGTAACAATAACGTTATAAATTTGGTCATCGCCCAGAAGTGATCCGGGT
TGGCTTAGTTCGGCTCGAATGAGAAGGCTTAGGGCGGTTCCC

> hap13

CGGTCTGTAAGGAGAAATGAAATTACAGCAGCTAAAACTGGTAAGGATAAGAGAAGAAGTACGGCTGTTACG
AGCACAGCTCAAACAAAGAGAGGTGTTTGATATTGAGAGATGGCTGGTGGTTTTATATTAATAGTTGTGGTGA
TAAAGTTAATTGCTCCTAAAATTGATGACACACCTGCTAGGTGAAGAGAGAAAATTGTTAGGTCTACGGATGC
TCCTGCGTGGGCAAGATTGCCCGCGAGTGGTGGATAAACTGTTCATCCTGTTCCGGCCCCGGCCTCAACACCA
GAAGAGGCTAGTAGTAGAAGGAAAGATGGGGGTAGGAGTCAAAAGCTTATATTATTTATTCGTGGGAATGCT
ATATCAGGTGCCCCAATCATTAGGGGTACGAGTCAGTTTCCAAACCCTCCAATAAGGATCGGTATTACTATAAA
GAAAATTATTACGAAGGCATGGGCAGTAACAATAACATTATAAATTTGGTCATCACCCAGAAGTGATCCGGGT
TGGCTTAGTTCGGCTCGAATGAGAAGGCTTAGGGCGGTTCCC

> hap14

CGGTCTGTAAGGAGTAATGAAATTCCAGCAGCTAAAACTGGTAAGGATAAGACAACAAGTACGGCTGTTACG
AGCACAGCTCAAACAAAGAGAGGTGTTTGATATTGAGAGATGGCTGGTGGTTTTATATTAATAGTTGTGGTGA
TAAAGTTAATTGCTCCTAAAATTGATGACACACCTGCTAGGTGAAGAGAGAAAATTGTTAGGTCTACGGATGC
TCCTGCGTGGGCAAGATTGCCCGCGAGTGGTGGATAAACTGTTCATCCTGTTCCGGCCCCGGCCTCAACACCA
GAAGAGGCTAGTAGTAGAAGGAAAGATGGGGGTAGGAGTCAAAAGCTTATATTATTTATTCGTGGGAATGCT
ATATCAGGTGCCCCAATCATTAGGGGTACGAGTCAGTTTCCAAACCCTCCAATAAGGATTGGTATTACTATAAA
GAAAATTATTACGAAGGCATGGGCAGTAACAATAACATTATAAATTTGGTCATCACCCAGAAGTGATCCGGGT
TGGCTTAGTTCGGCTCGAATGAGAAGGCTTAGGGCGGTTCCC

> hap15

CGGTCTGTAAGGAGTAAAGTAATTCCAGCAGCTAAAACTGGTAAGGATAAGAGAAGAAGTACGGCTGTTACG
AGCACAGCTCAAACAAAGAGAGGTGTTTGATATTGAGAGATGGCTGGTGGTTTTATATTAATAGTTGTGGTGA
TAAAGTTAATTGCTCCTAAAATTGATGACACACCTGCTAGGTGAAGAGAGAAAATTGTTAGGTCTACGGATGC
TCCTGCGTGGGCAAGATTGCCCGCGAGTGGTGGATAAACTGTTCATCCTGTTCCGGCCCCGGCCTCAACACCA
GAAGAGGCTAGTAGTAGAAGGAAAGATGGGGGTAGGAGTCAAAAGCTTATATTATTTATTCGTGGGAATGCT
ATATCAGGTGCTCCAATCATTAGGGGTACGAGTCAGTTTCCAAACCCTCCAATAAGGATTGGTATTACTATAAA
GAAAATTATTACGAAGGCATGGGCAGTAACAATAACATTATAAATTTGGTCATCACCCAGAAGTGATCCGGGT
TGGCTTAGTTCGGCTCGAATGAGAAGGCTTAGGGCGGTTCCC

> hap16

CGGTCTGTAAGGAGTAATGAAATTCCAGCAGCTAAAACTGGTAAGGATAAGAGAAGAAGTACGGCTGTTACG
AGCACAGCTCAAACAAAGAGAGGTGTTTGATATTGAGAGATGGCTGGTGGTTTTATATTAATAGTTGTGGTGA
TGAAGTTAATTGCTCCTAAAATTGATGACACACCTGCTAGGTGAAGAGAGAAAATTGTTAGGTCTACGGATGC
TCCTGCGTGGGCAAGATTGCCCGCGAGTGGTGGATAAACTGTTCATCCTGTTCCGGCCCCGGCCTCAACACCA
GAAGAGGCTAGTAGTAGAAGGAAAGATGGGGGTAGGAGTCAAAAGCTTATATTATTTATTCGTGGGAATGCT
ATATCAGGTGCTCCAATCATTAGGGGCACGAGTCAGTTTCCAAACCCTCCAATAAGGATTGGTATTACTATAAA
GAAAATTATTACGAAGGCATGGGCAGTAACAATAACATTATAAATTTGGTCATCACCCAGAAGTGATCCGGGT
TGGCTTAGTTCGGCTCGAATGAGAAGGCTTAGGGCGGTTCCC

> hap17

CGGTCTGTAAGGAGTAATGTAATTCCAGCAGCTAAAACTGGTAAGGATAAGAGAAGAAGTACGGCTGTTACG
AGCACAGCTCAAACAAAGAGAGGTGTTTGATATTGAGAGATGGCTGGTGGTTTTATATTAATAGTTGTGGTGA
TAAAGTTAATTGCTCCTAAAATTGATGACACACCTGCTAGGTGAAGAGAGAAAATTGTTAGGTCTACGGATGC
TCCTGCGTGGGCAAGATTGCCCGCGAGTGGTGGATAAACTGTTCATCCTGTTCCGGCCCCGGCCTCAACACCA
GAAGAGGCTAGTAGTAGAAGGAAAGATGGGGGTAGGAGTCAAAAGCTTATATTATTTATTCGTGGGAATGCT
ATATCAGGTGCCCCAATCATTAGGGGTACGAGTCAGTTTCCAAACCCTCCAATAAGGATTGGTATTACTATAAA
GAAAATTATTACGAAGGCATGGGCAGTAACAATAACATTATAAATTTGGTCATCACCCAGAAGTGATCCGGGT
TGGCTTAGTTCGGCTCGAATGAGAAGGCTTAGGGCGGTTCCC

> hap18

CGGTCTGTAAGGAGTAATGTAATTCCAGCAGCTAAAACTGGTAAGGATAAGAGAAGAAGTACGGCTGTTACG
AGCACAGCTCAAACAAAGAGAGGTGTTTGATATTGAGAGATGGCTGGTGGTTTTATATTAATAGTTGTGGTGA
TGAAGTTAATTGCTCCTAAAATTGATGACACACCTGCTAGGTGAAGAGAGAAAATTGTTAGGTCTACGGATGC
TCCTGCGTGGGCAAGATTGCCCGCGAGTGGTGGATAAACTGTTCATCCTGTTCCGGCCCCGGCCTCAACACCA
GAAGAGGCTAGTAGTAGAAGGAAAGATGGGGGTAGGAGTCAAAAGCTTATATTATTTATTCGTGGGAATGCT
ATATCAGGTGCTCCAATCATTAGGGGCACGAGTCAGTTTCCAAACCCTCCAATAAGGATTGGTATTACTATAAA

GAAAATTATTACGAAGGCATGGGCAGTAACAATAACATTATAAATTTGGTCATCACCCAGAAGTGATCCGGGT
TGGCTTAGTTCGGCTCGAATGAGAAGGCTTAGGGCGGTTCCC

> hap19

CGGTCTGTAAGGAGTATGGTAATTCCAGCAGCTAAAACTGGTAAGGATAAGAGAAGAAGTACGGCTGTTACG
AGCACAGCTCAAACAAAGAGAGGTGTTTGATATTGAGAGATGGCTGGTGGTTTTATATTAATAGTTGTGGTGA
TAAAGTTAATTGCTCCTAAAATTGATGACACACCTGCTAGGTGAAGAGAGAAAATTGTTAGGTCTACGGATGC
TCCTGCGTGGGCAAGATTGCCCGCGAGTGGTGGATAAACTGTTCATCCTGTTCCGGCCCCGGCCTCAACACCA
GAAGAGGCTAGTAGTAGAAGGAAAGATGGGGGTAGGAGTCAAAAGCTTATATTATTTATTCGTGGGAATGCT
ATATCAGGTGCCCCAATCATTAGGGGTACGAGTCAGTTTCCAAACCCTCCAATAAGGATTGGTATTACTATAAA
GAAAATTATTACGAAGGCATGGGCAGTAACAATAACATTATAAATTTGGTCATCACCCAGAAGTGATCCGGGT
TGGCTTAGTTCGGCTCGAATGAGAAGGCTTAGGGCGGTTCCC

> hap20

CGGTCTGTAAGGAGTAAAGTAATTCCAGCAGCTAAAACTGGTAAGGATAAGAGAAGAAGTACGGCTGTTACG
AGCACAGCTCAAACAAAGAGAGGTGTTTGATATTGAGAGATGGCTGGTGGTTTTATATTAATAGTTGTGGTGA
TGAAGTTAATTGCTCCTAAAATTGATGACACACCTGCTAGGTGAAGAGAGAAAATTGTTAGGTCTACGGATGC
TCCTGCGTGGGCAAGATTGCCCGCGAGTGGTGGATAAACTGTTCATCCTGTTCCGGCCCCGGCCTCAACACCA
GAAGAGGCTAGTAGTAGAAGGAAAGATGGGGGTAGGAGTCAAAAGCTTATATTATTTATTCGTGGGAATGCT
ATATCAGGTGCTCCAATCATTAGGGGCACGAGTCAGTTTCCAAACCCTCCAATAAGGATTGGTATTACTATAAA
GAAAATTATTACGAAGGCATGGGCAGTAACAATAACATTATAAATTTGGTCATCACCCAGAAGTGATCCGGGT
TGGCTTAGTTCGGCTCGAATGAGAAGGCTTAGGGCGGTTCCC

> hap21

CGATCTGTAAGGAGTATTGTAATTCCAGCAGCTAAAACTGGTAAGGATAAGAGAAGAAGTACGGGTGTTACG
AGCACAGCTCAAACAAAGAGAGGTGTTTGATATTGAGAGATGGCTGGTGGTTTTATATTAATAGTTGTGGTGA
TGAAGTTAATTGCTCCTAAAATTGATGACACACCTGCTAGGTGAAGAGAGAAAATTGTTAGGTCTACGGATGC
TCCTGCGTGGGCAAGATTGCCCGCGAGTGGTGGATAAACTGTTCATCCTGTTCCGGCCCCGGCCTCAACACCA
GAAGAGGCTAGTAGTAGAAGGAAAGATGGGGGTAGGAGTCAAAAGCTTATATTATTTATTCGTGGGAATGCT
ATATCAGGTGCCCCAATCATTAGGGGCACGAGTCAGTTTCCAAACCCTCCAATAAGGATTGGTATTACTATAAA
GAAAATTATTACGAAGGCATGGGCAGTAACAATAACATTATAAATTTGGTCATCACCCAGAAGTGATCCGGGT
TGGCTTAGTTCGGCTCGAATGAGAAGGCTTAGGGCGGTTCCC

> hap22

CGGTCTGTAAGGAGTAAAGTAATTCCAGCAGCTAAAACTGGTAAGGATAAGAGAAGAAGTACGGCTGTTACG
AGCACAGCTCAAACAAAGAGAGGTGTTTGATATTGAGAGATGGCTGGTGGTTTTATATTAATAGTTGTGGTGA
TAAAGTTAATTGCTCCTAAAATTGATGACACACCTGCTAGGTGAAGAGAGAAAATTGTTAGGTCTACGGATGC
TCCTGCGTGGGCAAGATTGCCCGCGAGTGGTGGATAAACTGTTCATCCTGTTCCGGCCCCGGCCTCAACACCA
GAAGAGGCTAGTAGTAGAAGGAAAGATGGGGGTAGGAGTCAAAAGCTTATATTATTTATTCGTGGGAATGCT
ATATCAGGTGCCCCAATCATTAGGGGTACGAGTCAGTTTCCAAACCCTCCAATAAGGATCGGTATTACTATAAA
GAAAATTATTACGAAGGCATGGGCAGTAACAATAACATTATAAATTTGGTCATCACCCAGAAGTGATCCGGGT
TGGCTTAGTTCGGCTCGAATGAGAAGGCTTAGGGCGGTTCCC

> hap23

CGGTCTGTAAGAAGTATTGTAATTCCAGCAGCTAAAACTGGTAAGGATAAGAGAAGAAGTACGGCTGTTACG
AGCACAGCTCAAACAAAGAGAGGTGTTTGATATTGAGAGATGGCTGGTGGTTTTATATTAATAGTTGTGGTGA
TAAAGTTAATTGCTCCTAAAATTGATGACACACCTGCTAGGTGAAGAGAGAAAATTGTTAGGTCTACGGATGC

TCCTGCGTGGGCAAGATTGCCCGCGAGTGGTGGATAAACTGTTCATCCTGTTCCGGCCCCGGCCTCAACACCA
GAAGAGGCTAGTAGTAGAAGGAAAGATGGGGGTAGGAGTCAAAAGCTTATATTATTTATTCGTGGGAATGCT
ATATCAGGTGCCCCAATCATTAGGGGTACGAGTCAGTTTCCAAACCCTCCAATAAGGATCGGTATTACTATAAA
GAAAATTATTACGAAGGCATGGGCAGTAACAATAACATTATAAATTTGGTCATCACCCAGAAGTGATCCGGGT
TGGCTTAGTTCGGCTCGAATGAGAAGGCTTAGGGCGGTTCCC

> hap24

CGGTCTGTAAGGAGTAATGTAATTCCAGCAGCTAAAACTGGTAAGGATAAGAGAAGAAGTACGGCTGTTACG
AGCACAGCTCAAACAAAGAGAGGTGTTTGATATTGAGAGATGGCTGGTGGTTTTATATTAATAGTTGTGGTGA
TAAAGTTAATTGCTCCTAAAATTGATGACACACCTGCTAGGTGAAGAGAGAAAATTGTTAGGTCTACGGATGC
TCCTGCGTGGGCAAGATTGCCCGCGAGTGGTGGATAAACTGTTCATCCTGTTCCGGCCCCGGCCTCAACACCA
GAAGAGGCTAGTAGTAGAAGGAAAGATGGGGGTAGGAGTCAAAAGCTTATATTATTTATTCGTGGGAATGCT
ATATCAGGTGCTCCAATCATTAGGGGTACGAGTCAGTTTCCAAACCCTCCAATAAGGATTGGTATTACTATAAA
GAAAATTATTACGAAGGCATGGGCAGTAACAATAACATTATAAATTTGGTCATCACCCAGAAGTGATCCGGGT
TGGCTTAGTTCGGCTCGAATGAGAAGGCTTAGGGCGGTTCCC

> hap25

CGGTCTGTAAGGAGTAATGTAATTCCAGCAGCTAAAACTGGTAAGGATAAGAGAAGAAGTACGGCTGTTACG
AGCACAGCTCAAACAAAGAGAGGTGTTTGATATTGAGAGATGGCTGGTGGTTTTATATTAATAGTTGTGGTGA
TAAAGTTAATTGCTCCTAAAATTGATGACACACCTGCTAGGTGAAGAGAGAAAATTGTTAGGTCTACGGATGC
TCCTGCGTGGGCAAGATTGCCCGCGAGTGGTGGATAAACTGTTCATCCTGTTCCGGCCCCGGCCTCAACACCA
GAAGAGGCTAGTAGTAGAAGGAAAGATGGGGGTAGGAGTCAAAAGCTTATATTATTTATTCGTGGGAATGCT
ATATCAGGTGCCCCAATCATTAGGGGTACGAGTCAGTTTCCAAACCCTCCAATAAGGATCGGTATTACTATAAA
GAAAATTATTACGAAGGCATGGGCAGTAACAATAACATTATAAATTTGGTCATCACCCAGAAGTGATCCGGGT
TGGCTTAGTTCGGCTCGAATGACAAGGCTTATGGCGGTTCCC

> hap26

CGGTCTGTAAGGAGTAATGTAATTCCAGCAGCTAAAACTGGTAAGGATAACAGAAGAAAAACGGCTGTTACG
AGCACAGCTCAAACAAAGAGAGGTGTTTGATATTGAGAGCCGGCTGGTGGTTTTATATTAATAGTTGTGGTGA
TAAAGTTAATTGCTCCTAAAATTGATGACACACCTGCTAGGTGAAGAGAGAAAATTGTTAGGTCTACGGATGC
TCCTGCGTGGGCAAGATTGCCCGCGAGTGGTGGATAAACTGTTCATCCTGTTCCGGCCCCGGCCTCAACACCA
GAAGAGGCTAGTAGCAGAAGGAAAGATGGGGGTAGGAGTCAAAAGCTTATATTATTTATTCGTGGGAATGCT
ATATCAGGTGCCCCAATCATTAGGGGTACGAGTCAGTTCCCAAACCCTCCAATAAGGATCGGCATTACTATAAA
GAAAATTATTACGAAGGCATGGGCAGTAACAATAACATTATAAATTTGGTCATCACCCAGAAGTGATCCGGGT
TGGCTTCCCTCGGCTCGAATGAGAAGGCTTAGGGCGGTTCCC

> hap27

CGGTCTGTAAGGAGGAATGTAATTCCAGCAGCTAAAACTGGTAAGGATAAGAGAAGAAGTACGGCTGTTACG
AGCACAGCTCAAACAAAGAGAGGTGTTTGATATTGAGAGATGGCTGGTGGTTTTATATTAATAGTTGTGGTGA
TGAAGTTAATTGCTCCTAAAATTGATGACACACCTGCTAGGTGAAGAGAGAAAATTGTTAGGTCTACGGATGC
TCCTGCGTGGGCAAGATTGCCCGCGAGTGGTGGATAAACTGTTCATCCTGTTCCGGCCCCGGCCTCAACACCA
GAAGAGGCTAGTAGTAGAAGGAAAGATGGGGGTAGGAGTCAAAAGCTTATATTATTTATTCGTGGGAATGCT
ATATCAGGTGCCCCAATCATTAGGGGCACGAGTCAGTTTCCAAACCCTCCAATAAGGATTGGTATTACTATAAA
GAAAATTATTACGAAGGCATGGGCAGTAACAATAACATTATAAATTTGGTCATCACCCAGAAGTGATCCGGGT
TGGCTTAGTTCGGCTCGAATGAGAAGGCTTAGGGCGGTTCCC

> hap28

CGGTCTGTAAGGAGTAATGTAATTCCAGCAGCTAAAACTGGTAAGGATAAGAGAAGAAGTACGGCTGTTACG
AGCACAGCTCAAACAAAGAGAGGTGTTTGATATTGAGAGATGGCTGGTGGTTTTATATTAATAGTTGTGGTGA
TAAAGTTAATTGCTCCTAAAATTGATGACACACCTGCTAGGTGAAGAGAGAAAATTGTTAGGTCTACGGATGC
TCCTGCGTGGGCAAGATTGCCCGCGAGTGGTGGATAAACTGTTCATCCTGTTCCGGCCCCGGCCTCAACACCA
GAAGAGGCTAGTAGTAGAAGGAAAGATGGGGGTAGGAGTCAAAAGCTTATATTATTTATTCGTGGGAATGCT
ATATCAGGTGCCCCAATCATTAGGGGTACGAGTCAGTTTCCAAACCCTCCAATAAGGATCGGTATTACTATAAA
GAAAATTATTACGAAGGCATGGGCAGTAACAATAACATTATAAATTTGGTCATCACCCAGAAGTGATCCGGGC
CGCCTTAGTTCGGCTCGAATGAGAAGGCTTAGCGCGGTTCCC

> hap29

CGATCTGTAAGGAGTATTGTAATTCCAGCAGCTAAAACTGGTAAGGATAACAGCAGAAGTACGGCTGTTACGA
GCACAGCTCAAACAAAGAGAGGTGTTTGATATTGAGAGATGGCTGGTGGTTTTATATTAATAGTTGTGGTGAT
GAAGTTAATTGCTCCTAAAATTGATGACACACCTGCTAGGTGAAGAGAGAAAATTGTTAGGTCTACGGATGCT
CCTGCGTGGGCAAGATTGCCCGCGAGTGGTGGATAAACTGTTCATCCTGTTCCGGCCCCGGCCTCAACACCAG
AAGAGGCTAGTAGTAGAAGGAAAGATGGGGGTAGGAGTCAAAAGCTTATATTATTTATTCGTGGGAATGCTA
TATCAGGTGCTCCAATCATTAGGGGCACGAGTCAGTTTCCAAACCCTCCAATAAGGATTGGTATTACTATAAAG
AAAATTATTACGAAGGCATGGGCAGTAACAATAACATTATAAATTTGGTCATCACCCAGAAGTGATCCGGGTT
GGCTTAGTTCGGCTCGAATGAGAAGGCTTAGGGCGGTTCCC

> hap30

CGGTCTGTAAGGAGTATTGTAATTCCAGCAGCTAAAACTGGTAAGGATAAGAGAAGAAGTACGGCCGTTACA
AGCACAGCTCAAACAAAGAGAGGTGTTTGATATTGGGAAATGGCTGGTGGTTTTATGTTAATAGTTGTGGTGA
TGAAGTTAATTGCCCCTAAAATTGATGATACACCTGCTAAGTGGAGGGAGAAAATTGTTAGGTCTACGGATGC
TCCTGCGTGAGCAAGATTACCCGCGAGTGGCGGGTAAACTGTTCATCCTGTTCCGGCCCCGGCCTCGACACCA
GAAGAGGCTAGTAGTAGAAGGAAAGAGGGGGGCAGGAGTCAAAAGCTTATATTATTTATTCGTGGGAATGCT
ATATCAGGTGCTCCAATCATTAGTGGCACGAGTCAGTTTCCAAATCCTCCAATAAGGATTGGTATCACTATAAA
GAAAATTATTACGAAGGCATGGGCAGTAACAATAACGTTATAAATTTGGTCATCGCCCAGAAGTGATCCGGGT
TGGCTTAGTTCGGCTCGAATGAGAAGGCTTAGGGCGGTTCCC

> hap31

CGGTCTGTAAGGAGTATTGTAATTCCAGCAGCTAAAACTGGTAAGGATAAGAGAAAAAGTACGGCTGTTACG
AGCACAGCTCAAACAAAGAGAGGTGTTTGATATTGAGAGATGGCTGGTGGTTTTATATTAATAGTTGTGGTGA
TAAAGTTAATTGCTCCTAAAATTGATGACACACCTGCTAGGTGAAGAGAGAAAATTGTTAGGTCTACGGATGC
TCCTGCGTGGGCAAGATTGCCCGCGAGTGGTGGATAAACTGTTCATCCTGTTCCGGCCCCGGCCTCAACACCA
GAAGAGGCTAGTACTAGAAGGAAAGATGGGGGTAGGAGTCAAAAGCTTATATTATTTATTCGTGGGAATGCT
ATATCAGGTGCCCCAATCATTAGGGGTACAAGGCAGTTTCCAAACCCTCCAATAAGGATCGGTATTACTATAAA
GAAAATTATTACGAAGGCATGGGCAGTAACAATAACATTATAAATTTGGTCATCACCCAGAACTGATCCGGGT
TGGCTTAGCTCGGCTCGAATGTGAAGGCTTAGGGCGGTTCCC

> hap32

CGGTCTGTAAGGAGTATTGTAATTCCAGCAGCTAAAACTGGTAAGGATAATAGAAGAAGTACGGCTGTTACGA
GCACAGCTCAAACAAAGAGAGGTGTTTGATATTGAGAGATGGCTGGTGGTTTTATATTAATAGTTGTGGTGAT
AAAGTTAATTGCTCCTAAAATTGATGACACACCTGCTAGGTGAAGAGAGAAAATTGTTAGGTCTACGGATGCT
CCTGCGTGGGCAAGATTGCCCGCGAGTGGTGGATAAACTGTTCATCCTGTTCCGGCCCCGGCCTCAACACCAG
AAGAGGCTAGTAGTAGAAGGAAAGATGGGGGTAGGAGTCAAAAGCTTATATTATTTATTCGTGGGAATGCTA
TATCAGGTGCCCCAATCATTAGGGGTACGAGTCAGTTTCCAAACCCTCCAATAAGGATTGGTATTACTATAAAG

AAAATTATTACGAAGGCATGGGCAGTAACAATAACATTATAAATTTGGTCATCACCCAGAAGTGATCCGGGTT
GGCTTAGTTCGGCTCGAATGAGAGGGCTTAGGGCGGTTCCC

> hap33

CGGTCTGTAAGGAGTATTGTAATTCCAGCAGCTAAAACTGGTAAGGATAAGAGAAGAAGTACGGCTGTTACG
AGCACAGCTCAAACAAAGAGAGGTGTTTGATATTGAGAGATGGCTGGTGGTTTTATATTAATAGTTGTGGTGA
TGAAGTTAATTGCTCCTAAAATTGATGACACACCTGCTAGGTGAAGAGAGAAAATTGTTAGGTCTACGGATGC
TCCTGCGTGGGCAAGATTGCCCGCGAGTGGTGGATAAACTGTTCATCCTGTTCCGGCCCCGGCCTCAACACCA
GAAGAGGCTAGTAGTAGAAGGAAAGATGGGGGTAGGAGTCAAAAGCTTATATTATTTATTCGTGGGAATGCT
ATATCAGGTGCTCCAATCATTAGGGGCACGAGTCAGTTTCCAAACCCTCCAATAAGGATTGGTATTACTATAAA
GAAAATTATTACGAAGGCATGGGCAGTAACAATAACATTATAAATTTGGTCATCACCCAGAAGTGATCCCGGG
TGGCTTAGTTCGGCTCGAATGAGAAGGCTTAGGGCGGTTCCC

> hap34

CGGTCTGTAAGGAGTATAGTAATTCCAGCAGCTAAAACTGGTAAGGATAAGAGAAGAAGTACGGCTGTTACG
AGCACAGCTCAAACAAAGAGAGGTGTTTGATATTGAGAGATGGCTGGTGGTTTTATATTAATAGTTGTGGTGA
TAAAGTTAATTGCTCCTAAAATTGATGACACACCTGCTAGGTGAAGAGAGAAAATTGTTAGGTCTACGGATGC
TCCTGCGTGGGCAAGATTGCCCGCGAGTGGTGGATAAACTGTTCATCCTGTTCCGGCCCCGGCCTCAACACCA
GAAGAGGCTAGTAGTAGAAGGAAAGATGGGGGTAGGAGTCAAAAGCTTATATTATTTATTCGTGGGAATGCT
ATATCAGGTGCCCCAATCATTAGGGGTACGAGTCAGTTTCCAAACCCTCCAATAAGGATCGGTATTACTATAAA
GAAAATTATTACGAAGGCATGGGCAGTAACAATAACATTATAAATTTGGTCATCACCCAGAAGTGATCCGGGT
TGGCTTAGTTCGGCTCGAATGAGAAGGCTTAGGGCGGTTCCC

> hap35

CGGTCTGTAAGGAGTATTGTAATTCCAGCAGCTAAAACTGGTAAGGATAGGAGAAGAAGTACGGCCGTTACA
AGCACAGCTCAAACAAAGAGAGGTGTTTGATATTGGGAAATGGCTGGTGGTTTTATGTTAATAGTTGTGGTGA
TGAAGTTAATTGCCCCTAAAATTGATGATACACCTGCTAAGTGGAGGGAGAAAATTGTTAGGTCTACGGATGC
TCCTGCGTGAGCAAGATTACCCGCGAGTGGCGGGTAAACTGTTCATCCTGTTCCGGCCCCGGCCTCGACACCA
GAAGAGGCTAGTAGTAGAAGGAAAGAGGGGGGCAGGAGTCAAAAGCTTATATTATTTATTCGTGGGAATGCT
ATATCAGGTGCTCCAATCATTAGTGGCACGAGTCAGTTTCCAAATCCTCCAATAAGGATTGGTATCACTATAAA
GAAAATTATTACGAAGGCATGGGCAGTAACAATAACGTTATAAATTTGGTCATCGCCCAGAAGTGATCCGGGT
TGGCTTAGTTCGGCTCGAATGAGAAGGCTTAGGGCGGTCCCC

> hap36

CGGTCTGTAAGGAGTATTGTAATTCCAGCAGCTAAAACTGGTAAGGATAAGAGAAGAAGTACGGCTGTTACG
AGCACAGCTCAAACAAAGAGAGGTGTTTGATATTGAGAGATGGCTGGTGGTTTTATATTAATAGTTGTGGTGA
TAAAGTTAATTGCTCCTAAAATTGATGACACACCTGCTAGGTGAAGAGAGAAAATTGTTAGGTCTACGGATGC
TCCTGCGTGGGCAAGATTGCCCGCGAGTGGTGGATAAACTGTTCATCCTGTTCCGGCCCCGGCCTCAACACCA
GAAGAGGCTAGTAGTAGAAGGAAAGATGGGGGTAGGAGTCAAAAGCTTATATTATTTATTCGTGGGAATGCT
ATATCAGGTGCCCCAATCATTAGGGGTACGAGTCAGTTTCCAAACCCTCCAATAAGGATCGGTATTACTATAAA
GAAAATTATTACGAAGGCTGGGGCAGTAACAATAACATTATAAATTTGGTCATCACCCAGAAGTGATCCGGGT
TGGCTTAGTTCGGCTCGAATGAGAAGGCTTAGGGCGGTTCCC

> hap37

CGGTCTGTAAGGAGTATTGTAATTCCAGCAGCTAAAACTGGTAAGGATAAGAGAAGAAGTACGGCTGTTACG
AGCACAGCTCAAACAAAGAGAGGTGTTTGATATTGAGAGATGGCTGGTGGTTTTATATTAATAGTTGTGGTGA
TGAAGTTAATTGCTCCTAAAATTGATGACACACCTGCTAGGTGAAGAGAGAAAATTGTTAGGTCTACGGATGC

TCCTGCGTGGGCAAGATTGCCCGCGAGTGGTGGATAAACTGTTCATCCTGTTCCGGCCCCGGCCTCAACACCA
GAAGAGGCTAGTAGTAGAAGGAAAGATGGGGGTAGGAGTCAAAAGCTTATATTATTTATTCGTGGGAATGCT
ATATCAGGTGCTCCAATCATTAGGGGCACGAGTCAGTTTCCAAACCCTCCAATAAGGATTGGTATTACTATAAA
GAAAATTATTACGAAGGCATGGGCAGTAACAATAACATTATAAATTTGGTCATCACCCAGAAGTGATCCGGGT
TGGCTTAGTTCGGCTCGAATGAGAAAGCTTAGGGCGGTTCCC

> hap38

CGGTCTGTAAGGAGTATTGTAATTCCAGCAGCTAAAACTGGTAAGGATAAGAGAAGAAGTACGGCTGTTACG
AGCACAGCTCAAACAAAGAGAGGTGTTTGATATTGAGAGATGGCTGGTGGTTTTATATTAATAGTTGTGGTGA
TGAAGTTAATTGCTCCTAAAATTGATGACACACCTGCTAGGTGAAGAGAGAAAATTGTTAGGTCTACGGATGC
TCCTGCGTGGGCAAGATTGCCCGCGAGTGGTGGATAAACTGTTCATCCTGTTCCGGCCCCGGCCTCAACACCA
GAAGAGGCTAGTAGTAGAAGGAAAGATGGGGGTAGGAGTCAAAAGCTTATATTATTTATTCGTGGGAATGCT
ATATCAGGTGCTCCAATCATTAGGGGCACGAGTCATTTTCCAAACCCTCCAATAAGGATTGGTATTACTATAAA
GAAAATTATTACGAAGGCATGGGCAGTACCAATAACATTATAAATTTGGTCATCACCCAGAATTGATCCGGGTT
GGCTTAGGTCGGCTCGAATGAGAAAGCTTAGGGGCGTTCCC

> hap39

CGGTCTGTAAGGAGTATTGTAATTCCAGCAGCTAAAACTGGTAAGGATAAGAGAAGAAGTACGGCTGTTACG
AGCCCAGCTCAAACAAAGAGAGGTGCCCGATATTGAGAGATGGCTGGTGGTTTTATATTAATAGTTGTGGTGA
TGAAGTTAATTGCTCCTAAAATTGATGACACACCTGCTAGGTGAAGAGAGAAAATTGTTAGGTCTACGGATGC
TCCTGCGTGGGCAAGATTGCCCGCGAGTGGTGGATAAACTGTTCATCCTGTTCCGGCCCCGGCCTCAACACCA
GAAGAGGCTAGTAGTAGAAGGAAAGATGGGGGTAGGAGTCAAAAGCTTATATTATTTATTCGTGGGAATGCT
ATATCAGGTGCTCCAATCATTAGGGGCACGAGTCAGTTTCCAAACCCTCCAATAAGGATTGGTATTACTATAAA
GAAAATTATTACGAAGGCATGGGCAGTAACAATAACATTATAAATTTGGTCATCACCCAGAAGTGATCCGGGT
TGGCTTAGTTCGGCTCGAATGAGAAGGCTTAGGGCGGTTCCC

> hap40

CGGTCTGTAAGGAGTATTGTAATTCCAGCAGCTAAAACTGGTAAGGATAAGAGAAGAAGTACGGCTGTTACG
AGCACAGCTCAAACAAAGAGAGGTGTTTGATATTGAGAGACGGCTGGTGGTTTTATATTAATAGTTGTGGTGA
TAAAGTTAATTGCTCCTAAAATTGATGACACACCTGCTAGGTGAAGAGAGAAAATTGTTAGGTCTACGGATGC
TCCTGCGTGGGCAAGATTGCCCGCGAGTGGTGGATAAACTGTTCATCCTGTTCCGGCCCCGGCCTCAACACCA
GAAGAGGCTAGTAGTAGAAGGAAAGATGGGGGTAGGAGTCAAAAGCTTATATTATTTATTCGTGGGAATGCT
ATATCAGGGGCCCCAATCATTAGGGGTACAAGTCAGTTTCCAAACCCTCCAATAAGGATCGGTATTACTATAAA
GAAAATTATTACGAAGGCATGGGCAGTAACAATAACATTATAAATTTGGTCATCACCCAGAAGTGATCCGGGT
TGGCTTAGTTCGGCTCGAATGAGAAGGCTTAGGGCGGTTCCC

> hap41

CGGTCTGTAAGGAGTATTGTAATTCCAGCAGCTAAAACTGGTAAGGATAAGAGAAGAAGTACGGCCGTTACA
AGCACAGCTCAAACAAAGAGAGGTGTTTGATATTGGGAAATGGCTGGTGGTTTTATGTTAATAGTTGTGGTGA
TGAAGTTAATTGCCCCTAAAATTGATGATACACCTGCTAAGTGGAGGGAGAAAATTGTTAGGTCTACGGATGC
TCCTGCGTGAGCAAGATTACCCGCGAGTGGCGGGTAAACTGTTCATCCTGTTCCGGCCCCGGCCTCGACACCA
GAAGAGGCTAGTAGTAGAAGGAAAGAGGGGGGCAGGAGTCAAAAGCTTATATTATTTATTCGTGGGAATGCT
ATATCAGGTGCTCCAATCATTAGTGGCACGAGTCAGTTTCCAAATCCTCCAATAAGGATTGGTATCACTATAAA
GAAAATTATTACGAAGGCATGGGCAGTAACAATAACGTTATAAATTTGGTCATCGCCCAGAAGTGATCCGGGT
TGGCTTAGTTCGGCTCGAATGAGAAAGCTTAGGGCGGTTCCC

> hap42

CGGTCTGTAAGGAGTATTGTAATTCCAGCAGCTAAAACTGGTAAGGATAAGAGAAGAAGTACGGCTGTTACG
AGCACAGCTCAAACAAAGAGAGGTGTTTGATATTGAGAGATGGCTGGTGGTTTTATATTAATAGTTGTGGTGA
TAAAGTTAATTGCTCCTAAAATTGATGACACACCTGCTAGGTGAAGAGAGAAAATTGTTAGGTCTACCGATGCT
CCTGCGTGGGCAAGATTGCCCGCGAGTGGTGGATAAACTGTTCATCCTGTTCCGGCCCCGGCCTCAACACCAG
AATAAGCTAGTAATAAAAAGAAAGATGGGGGTAGGAATCAAAAGCTTATATTATTTATTCGTGGGAATGCTAT
ATCAGGTGCCCCAATCATTAAGGGTACAAGTCAGTTTCCAAACCCTCCAATAAGGATTGGTATTACTATAAAGA
AAATTATTACAAAAGCATGGGCAGTAACAATAACATTATAAATTTGGTCATCACCCATAAGTGATCCCGGTTGG
CTTAATTCGGCTCGAATGAAAAAGCTTAAGGCGGTTTCC

> hap43

CGGTCTGTAAGGAGTATTGTAATTCCAGCAGCTAAAACTGGTAAGGATAAGAGAAGAAGTACGGCCGTTACA
AGCACAGCTCAAACAAAGAGAGGTGTTTGATATTGGGAAATGGCTGGTGGTTTTATGTTAATAGTTGTGGTGA
TGAAGTTAATTGCCCCTAAAATTGATGATACACCTGCTAAGTGGAGGGAGAAAATTGTTAGGTCTACGGATGC
TCCTGCGTGAGCAAGATTACCCGCGAGTGGCGGGTAAACTGTTCATCCTGTTCCGGCCCCGGCCTCGACACCA
GAAGAGGCTAGTAGTAGAAGGAAAGAGGGGGGCAGGAGTCAAAAGCTTATATTATTTATTCGTGGGAATGCT
ATATCAGGTGCTCCAATCATTAGGGGCACGAGTCAGTTTCCAAATCCTCCAATAAGGATTGGTATCACTATAAA
GAAAATTATTACGAAGGCATGGGCAGTAACAATAACGTTATAAATTTGGTCATCGCCCAGAAGTGATCCGGGT
TGGCTTAGTTCGGCTCGAATGAGAAGGCTTAGGGCGGTTCCC

> hap44

CGGTCTGTAATGAGTATTGTAATTCCAGCAGGTAAAACTGGTAAGGATAAGAGAAGAAGTACGGCTGTTACG
AGCACAGCTCAAACAAAGAGAGGTGTTTGATATTGAGAGATGGGTGGTGGTTTTATATTAATAGTTGTGGTGA
TAAAGTTAATTGCTCCTAAAATTGATGACACACCTGCTAGGTGAAGAGAGAAAATTGTTAGGTCTACGGATGC
TCCTGCGTGGGCAAGATTGCCCGCGAGTGGGGGATAAACTGGTCATCCTGGTCCGGCCCCGGCCTCAACACCA
GAAGAGGCTAGTAGTAGAAGGAAAGATGGGGGTAGGAGTCAAAAGCTTATATTATTTATTCGTGGGAATGCT
ATATCAGGTGCCCCAATCATTAGGGGTACGAGTCAGTTTCCAAACCCTCCAATAAGGATTGGTATTACTATAAA
GAAAATTATTACGAAGGCATGGGCAGTAACAATAACATTATAAATTTGGTCATCACCCAGAAGTGATCCGGGT
TGGCTTAATTCGGCTCGAATGAGAAGGCTTAGGGCGGTTCCC

> hap45

CGGTCTGTAAGGAGTAATGTAATTCCAGCAGCTAAAACTGGTAAGGATAAGAGAAGAAGTACGGCTGTTACG
AGCACAGCTCAAACAAAGAGAGGTGTTTGATATTGAGAGATGGCTGGTGGTTTTATATTAATAGTTGTGGTGA
TAAAGTTAATTGCTCCTAAAATTGATGACACACCTGCTAGGTGAAGAGAGAAAATTGTTAGGTCTACGGATGC
TCCTGCGTGGGCAAGATTGCCCGCGAGTGGTGGATAAACTGTTCATCCTGTTCCGGCCCCGGCCTCAACACCA
GAAGAGGCTAGTAGTAGAAGGAAAGATGGGGGTAGGAGTCAAAAGCTTATATTATTTATTCGTGGGAATGCT
ATATCAGGTGCCCCAATCATTAGGGGTACGAGTCAGTTTCCAAACCCTCCAATAAGGATCGGTATTACTATAAA
GAAAATTATTACGAAGGCATGGGCAGTAACAATAACCTTATAAATTTGGTCATCACCCCAAAATGATCCGGGTT
GGCTTACTTCGGCTCGAAGGAGAAGGCTTAGGGCGGTTCCC

> hap46

CGGTCTGTAAGGAGTATTGAAATTCCAGCAGCTAAAACTGGTAAGGATAAGAGAAGAAGTACGGCTGTTACG
AGCACAGCTCAAACAAAGAGAGGTGTTTGATATTGAGAGATGGCTGGTGGTTTTATATTAATAGTTGTGGTGA
TAAAGTTAATTGCTCCTAAAATTGATGACACACCTGCTAGGTGAAGAGAGAAAATTGTTAGGTCTACGGATGC
TCCTGCGTGGGCAAGATTGCCCGCGAGTGGTGGATAAACTGTTCATCCTGTTCCGGCCCCGGCCTCAACACCA
GAAGAGGCTAGTAGTAGAAGGAAAGATGGGGGTAGGAGTCAAAAGCTTATATTATTTATTCGTGGGAATGCT
ATATCAGGTGCCCCAATCATTAGGGGTACGAGTCAGTTTCCAAACCCTCCAATAAGGATCGGTATTACTATAAA

GAAAATTATTACGAAGGCATGGGCAGTAACAATAACATTATAAATTTGGTCATCACCCAGAAGTGATCCGGGT
TGGCTTAGTTCGGCTCGAATGAGAAGGCTTAGGGCGGTTCCC

> hap47

CGGTCTGTAAGGAGTACAGATATTCCAGCAGCTAAAACTGGTAAGGATAGGAGAAGAAGTACGGCCGTTACA
AGCACAGCTCAAACAAAGAGAGGTGTTTGATATTGGGAAATGGCTGGTGGTTTTATGTTAATAGTTGTGGTGA
TGAAGTTAATTGCCCCTAAAATTGATGATACACCTGCTAAGTGGAGGGAGAAAATTGTTAGGTCTACGGATGC
TCCTGCGTGAGCAAGATTACCCGCGAGTGGCGGGTAAACTGTTCATCCTGTTCCGGCCCCGGCCTCGACACCA
GAAGAGGCTAGTAGTAGAAGGAAAGAGGGGGGCAGGAGTCAAAAGCTTATATTATTTATTCGTGGGAATGCT
ATATCAGGTGCTCCAATCATTAGTGGCACGAGTCAGTTTCCAAATCCTCCAATAAGGAGTGGTATCACTATAAA
GAAAATTATTACGAAGGCATGGGCAGTAACAATAACGTTATAAATTTGGTCATCGCCCAGAAGTGATCCGGGT
TGGCTTAGTTCGGCTCGAATGAGAAAGGTTAGGGCGGTTCCC

> hap48

CGGTCTGTAAGGAGTAATGTAATTCCAGCAGCTAAAACTGGTAAGGATAAGAGAACAAGTACGGCTGTTACG
AGCACAGCTCAAACAAAGAGAGGTGTTTGATATTGAGAGATGGCTGGTGGTTTTATATTAATAGTTGTGGTGA
TAAAGTTAATTGCTCCTAACATTGATGACACACCTGCTAGGTGAAGAGAGAAAATTGTTAGGTCTACGGATGC
TCCTGCGTGGGCAAGATTGCCCGCGAGTGGTGGATAAACTGTTCATCCTGTTCCGGCCCCGGCCTCAACACCA
GAAGAGGCTAGTAGTAGAAGGAAAGATGGGGGTAGGAGTCAAAAGCTTATATTATTTATTCGTGGGAATGCT
ATATCAGGTGCCCCAATCATTAGGGGTACGAGTCAGTTTCCAAACCCTCCAATAAGGATCGGTATTACTATAAA
GAAAATTATTACGAAGGCATGGGCAGTAACAATAACATTATAAATTTGGTCATCCCCCAGAAGTGATCCGGGT
TGGCTTAGTTCGGCTCGAATGAAAAGGCTTAAGGGCGTTCCC

> hap49

CGGGCTGTAAGGAGTATTGGTATTCCAGCATGTAAAACTGGTAAGGGTAAGAGAAAAAATACGGGTGTTACG
GACGCCGGTCAATAAAAAAAAGGTGGTTGATATTGGGAAAAGGGTGGGGGTTTTATATTAATAGTTGTGGTG
ATGAAGTTAATTGCTCCTAAAATTGATGACACACCTGGTAGGTGAAAAGGAAAAATTGGTAAGTCTACGTGTG
GTCCTGCGGGGGCATAATTGGCCGCGTAAGGGGGATAAACTGGTCCTTCTGGTCCGGGCCCGGGCTCCTCCG
CAAAAGAGGGTAAGAATAAAAGGAAAGATGGGGGGAGGAATTCAAAGCTTATATTATTTATTCGGGGGGAT
GGTATATCAGGGGCTCCCATCATTAAGGGCACGAGTCAGTTTCCCAACCCTCCAATAAGGATTGGTATTACTAT
AAAGAAAATTATTACGAAGAGATGGGCAGTGAACATAACTTTATAAATTTTGGCATCACCCAGAAGTGATCCC
GGTTGTCTTAATTCGGGCCGAATGAAAAGGGTTAAGGCGGTTTTC

> hap50

CGGTCTGTAAGGAGTAATGTAATTCCAGCAGCTAAAACTGGTAAGGATAAGAGAAAAAGTACGGCTGTTACG
AGCACAGCTCAAACAAAGAGAGGTGTTTGATATTGATAGATGGCTGGTGGTTTTATATTAATAGTTGTGGTGA
TAAAGTTAATTGCTCCTAAAATTGATGACACACCTGCTAGGTGAAGAGAGAAAATTGTTAGGTCTACGGATGC
TCCTGCGTGGGCAAGATTGCCCGCGAGTGGTGGATAAACTGTTCATCCTGTTCCGGCCCCGGCCTCAACACCA
GAAGAGGCTAGTAGTAGAAGGAAAGATGGGGGTAGGAGTCAAAAGCTTATATTATTTATTCGTGGGAATGCT
ATATCAGGTGCCCCAATCATTAAGGGTACGAGTCAGTTTCCAAACCCTCCAATAAGGATCGGTATTACTATAAA
GAAAATTATTACGAAGGCATGGGCAGTAACAATAACTTTATAAATTTGTTCTTCACCAAGAATTGATTCCGGTT
GGCTTAGTTCGGCTCGAATGAGAAGGCTTAGGGCGGTTCCC

> hap51

CGGTCTGTAAAGAGTATTGTAATTCCAGCAGCTAAAACTGGTAAGGATAAGAGAAGAAGTACGGCTGTTACG
AGCACAGCTCAAACAAAGAGAGGTGTTTGATATTGAGAGATGGCTGGTGGTTTTATATTAATAGTTGTGGTGA
TAAAGTTAATTGCTCCTAAAATTGATGACACACCTGCTAGGTGAAGAGAGAAAATTGTTAGGTCTACGGATGC

TCCTGCGTGGGCAAGATTGCCCGCGAGTGGTGGATAAACTGTTCATCCTGTTCCGGCCCCGGCCTCAACACCA
GAAGAGGCTAGTAGTAGAAGGAAAGATGGGGGTAGGAGTCAAAAGCTTATATTATTTATTCGTGGGAATGCT
ATATCAGGTGCCCCAATCATTAGGGGTACGAGTCAGTTTCCAAACCCTCCAATAAGGATCGGTATTACTATAAA
GAAAATTATTACGAAGGCATGGGCAGTAACAATAACATTATAAATTTGGTCATCACCCAGAAGTGATCCGGGT
TGGCTTAGTTCGGCTCGAATGAGAAGGCTTAGGGCGGTTCCC

> hap52

CGGTCTGTAAGGAGTAATGAAATTCCAGCAGCTAAAACTGGCAAGGATAAGAGAAGAAGGACGGCTGTTACC
ACCACACCTCAAACAAAGAGAGCTGTTTTTTATTGTTAGACGGCTGCCGGTTTTATATTAATAGTTGTGGTGAT
GAAGTTAATTGCTCCTAAAATTGATGACACACCTGCTAGGTGAAGAGAGAAAATTGTTAGGTCTACGGATGCT
CCTGCGTGGGCAAGATTGCCCGCGAGTGGTGGATAAACTGTTCATCCTGTTCCGGCCCCGGCCTCAACACCAG
AAGAGGCTAGTAGTAGAAGGAAAGATGGGGGTAGGAGCCAAAAGCTAATATTATTTATTCGGGGGAATGCTA
TATCAGGGCCTCCAATCATTAGGGGCACGAGTCAGTTTCCAACCCCTCCAATAAGGATTGGTATTACTATAAAG
AAAATTATTACGAAGCCATGGGCAGTAACAATAACATTATAAATTTGGCCATCACCCATAAGTGACCCGGGTTG
GCTTATTTCGGCTCGAATGGGAAGGCTTAAGGCCGCTCCC
