# Supplementary material for: Comparison of the Genetic Structure of Invasive Bigheaded Carp (Hypophthalmichthys spp.) Populations in Central-European Lacustrine and Riverine Habitats
Source: Animals (Basel). 2021 Jul 6;11(7):2018. doi: 10.3390/ani11072018 (PMC8300242; doi:10.3390/ani11072018)
Supplement: Supplementary file 1 [file animals-11-02018-s001.zip › Supplementary material/Table S3.pdf]

Table S3
Bootstrap 95% intervals for Fst and Cavalli-Sforza and Edwards genetic distance

Title: Comparison of the genetic structure of invasive bigheaded carp (Hypophthalmichthys spp.) populations in Central-European lacustrine and riverine habitats
Authors: Tamás Molnár *, István Lehoczky*, Erika Edviné Meleg, Gergely Boros, András Specziár, Attila Mozsár, Zoltán Vitál, Vilmos Józsa, Wahiba Allele, Béla Urbányi and Balázs Kovács

Bootstrap resampling over loci

95% Confidence Interval

Fst not using ENA

pop	1	2	3	4
2	-0.003384			
3	0.005719	0.014902		
4	0.188285	0.236275	0.244859	
5	0.051867	0.079888	0.053622	0.265114

	1	2	3	4
2	0.033932			
3	0.024600	0.063304		
4	0.240668	0.275970	0.313588	
5	0.222484	0.272011	0.241777	0.351813

Fst using ENA

pop	1	2	3	4
2	0.001086			
3	0.006160	0.015180		
4	0.174498	0.220465	0.222770	
5	0.047482	0.084994	0.052586	0.242486

	1	2	3	4
2	0.041572			
3	0.025741	0.063513		
4	0.240520	0.264023	0.307646	
5	0.223088	0.274284	0.243343	0.343550

Bootstrap resampling over loci

95% Confidence Interval

Dc not using INA

pop	1	2	3	4
2	0.302844			
3	0.291125	0.352710		
4	0.585466	0.622771	0.737144	
5	0.427774	0.524855	0.447962	0.781014

	1	2	3	4
2	0.449807			
3	0.398937	0.537124		
4	0.693136	0.796938	0.870182	
5	0.622633	0.702000	0.650808	0.879053

Dc using INA

pop	1	2	3	4
2	0.310019			
3	0.284522	0.354617		
4	0.590053	0.624643	0.734417	
5	0.434581	0.549034	0.465345	0.775461

	1	2	3	4
2	0.459289			
3	0.405719	0.540174		
4	0.690734	0.791501	0.870143	
5	0.623253	0.710678	0.659184	0.868765
