# Supplementary material for: Comparison of the Genetic Structure of Invasive Bigheaded Carp (Hypophthalmichthys spp.) Populations in Central-European Lacustrine and Riverine Habitats
Source: Animals (Basel). 2021 Jul 6;11(7):2018. doi: 10.3390/ani11072018 (PMC8300242; doi:10.3390/ani11072018)
Supplement: Supplementary file 1 [file animals-11-02018-s001.zip › Supplementary material/Table S4.pdf]

Table S4

The mean log likelihood data and Evanno’s delta K of the hierarchical STRUCTURE analysis

Title: Comparison of the genetic structure of invasive bigheaded carp (Hypophthalmichthys spp.) populations in Central-European lacustrine and riverine habitats

Authors: Tamás Molnár *, István Lehoczky*, Erika Edviné Meleg, Gergely Boros, András Specziár, Attila Mozsár, Zoltán Vitál, Vilmos Józsa, Wahiba Allele, Béla Urbányi and Balázs Kovács

First run

# K	Reps	Mean LnP(K)	Stdev LnP(K)	Ln'(K)	|Ln''(K)|	Delta K
1	10	-8597.7200	0.4590	NA	NA	NA
2	10	-7408.2700	0.5078	1189.450	746.24000	1469.475024
3	10	-6965.0600	14.7411	443.2100	101.60000	6.892288
4	10	-6623.4500	1.1038	341.6100	219.39000	198.762186
5	10	-6501.2300	77.3200	122.2200	10.890000	0.140843
6	10	-6368.1200	53.9098	133.1100	108.63000	2.015033
7	10	-6343.6400	123.6475	24.48000	47.050000	0.380517
8	10	-6366.2100	207.6794	-22.57000	NA	NA

Second run

# K	Reps	Mean LnP(K)	Stdev LnP(K)	Ln'(K)	|Ln''(K)|	Delta K
1	10	-6886.2200	0.3938	NA	NA	NA
2	10	-6400.7700	37.9274	485.4500	74.280000	1.958481
3	10	-5989.6000	44.9529	411.1700	86.110000	1.915562
4	10	-5664.5400	1.9879	325.0600	260.20000	130.8950
5	10	-5599.6800	10.2976	64.86000	NA	NA
